# Integrative genomics and experimental validation identify a mechanosensitive gene signature associated with colorectal cancer liver metastasis

**DOI:** 10.1016/j.isci.2026.117926

**Published:** 2026-10-16

**Authors:** Tianxue Yang, Ye Yuan, Xue Li, Jiaqi Wang, Chenyao Li, Ziran Xu

**Affiliations:** 1 Department of Clinical Laboratory, Lequn Branch, The First Hospital of Jilin University, Changchun, Jilin, China; 2 Department of Colorectal and Anal Surgery, General Surgery Center, The First Hospital of Jilin University, Changchun, Jilin, China; 3 Department of Medical Oncology, West German Cancer Center, University Hospital Essen, 45147 Essen, Germany; 4 Department of Thyroid Surgery, General Surgery Center, The First Hospital of Jilin University, Changchun, Jilin, China; 5 Department of Anorectal Surgery, 942 Hospital of Joint Logistic Support Unit, People’s Liberation Army, Yinchuan, China

**Keywords:** mechanical stimulation, colorectal cancer, liver metastasis, prognostic model, machine learning

## Abstract

The mechanical tumor microenvironment critically influences colorectal cancer (CRC) progression and metastasis, yet the underlying molecular mediators remain poorly defined. By integrating single-cell transcriptomics, bulk RNA-seq, weighted gene co-expression network analysis, and 117 machine learning algorithms, we identified a six-gene mechanosensitive signature (*SNX7*, *SDC1*, *CDCP1*, *PTGES*, *EMP2*, and *CALB2*) associated with CRC liver metastasis. This signature demonstrated superior prognostic performance (mean C-index 0.68) across multiple independent cohorts compared to 74 published CRC signatures. Among these genes, *SDC1* was consistently upregulated in CRC cells and tissues, and its expression was further induced by increased matrix stiffness in tunable hydrogel culture systems. These findings support a mechanosensitive gene signature with robust prognostic value and identify *SDC1* as a stiffness-responsive candidate regulator, providing insights into the mechanobiological regulation of CRC metastasis.

## Highlights

• Integrative analyses identify a six-gene mechanosensitive signature in colorectal cancer
• The signature is associated with prognosis and liver metastasis-related features
• Stiffness increases *SDC1* expression in RKO and Caco-2 cells
• *SDC1* knockdown reduces DLD-1 migration, invasion, paxillin, and vinculin levels

## Graphical abstract

**Figure:**
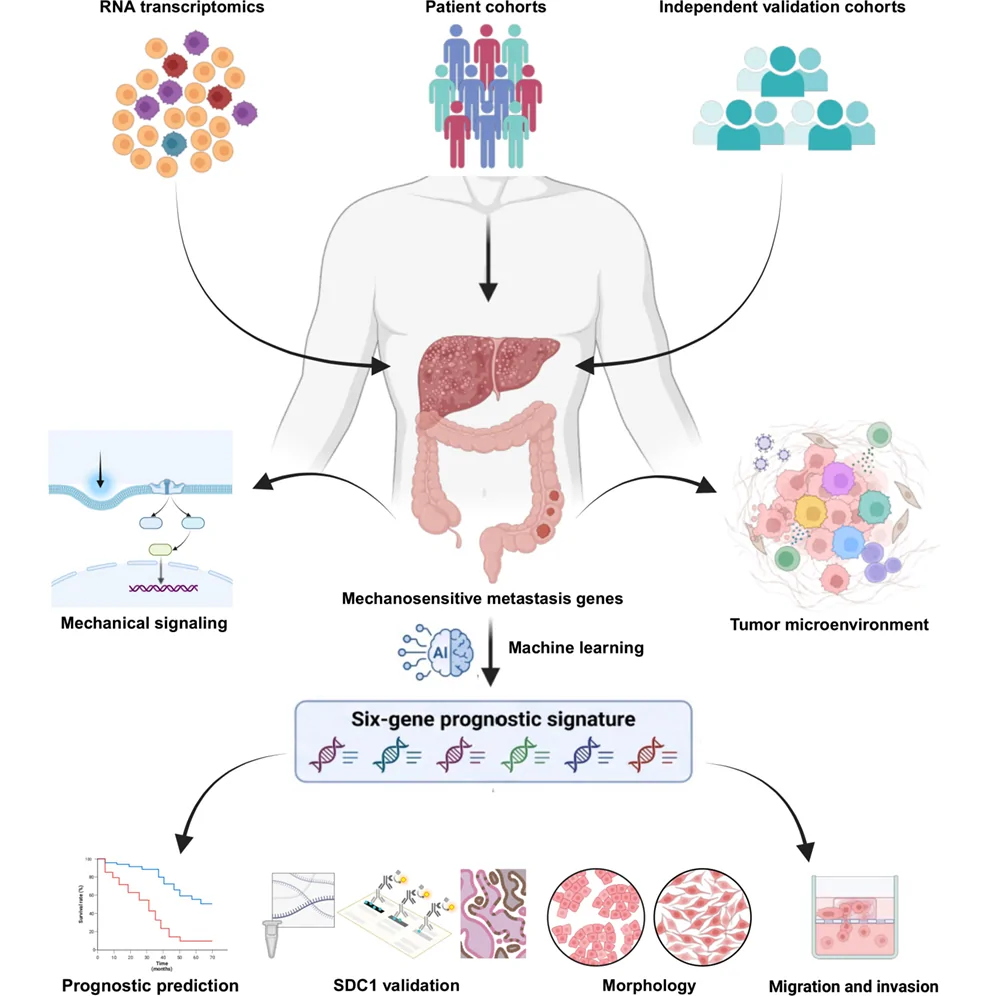

## Teaser

Genomics; Cell biology; Cancer

## Introduction

Colorectal cancer (CRC) remains a leading cause of cancer-related morbidity and mortality worldwide. In China, it ranks as the second most commonly diagnosed malignancy, and its incidence continues to rise due to factors such as population aging, dietary changes, and lifestyle shifts.^1^ Molecularly, the pathogenesis of CRC involves the synergistic effect of multiple key genes. Mutations in *APC* disrupt the homeostasis of the Wnt signaling pathway, leading to the formation of adenomatous polyps.^2^^,^^3^ Mutations in *KRAS* and *BRAF* promote tumor cell proliferation and invasion through activation of the MAPK and MEK/ERK signaling pathways.^4^^,^^5^ *TP53* mutations impair genomic surveillance mechanisms, accelerating malignant transformation,^6^^,^^7^ while *PIK3CA* mutations aberrantly activate the PI3K pathway, further enhancing tumor cell growth and survival.^8^^,^^9^ These genetic alterations collectively drive CRC development and progression, and influence treatment response and patient prognosis.^10^^,^^11^ As the disease advances, cancer cells can invade through the basement membrane into blood and lymphatic vessels, metastasizing to distant organs such as the liver and lungs, severely compromising organ function and reducing the 5-year survival rate to less than 20%.^12^^,^^13^ The high mortality associated with metastatic CRC underscores the urgent need to elucidate its molecular mechanisms. A deeper understanding may not only reveal the intrinsic drivers of invasion and metastasis but also provide a theoretical basis and technical support for early diagnosis, precision therapy, and targeted interventions, ultimately improving survival and quality of life for patients and having a profound impact on overall healthcare outcomes.

In oncology, mechanical stimulation has emerged as a pivotal and evolving research direction, revealing its multifaceted roles in the initiation and progression of solid tumors. Mechanical stimulation encompasses various physical forces within the cellular microenvironment, including matrix stiffness, shear stress, and compressive forces, all of which profoundly influence tumor cell behavior.^14^^,^^15^^,^^16^ Among these mechanical cues, the present study focuses primarily on matrix/substrate stiffness, because the experimental validation was performed using stiffness-tunable hydrogel culture systems. Accumulating evidence indicates that mechanical stimulation activates multiple signaling pathways that significantly affect tumor cell proliferation, metastasis, and invasion.^17^^,^^18^^,^^19^^,^^20^ In terms of proliferation, appropriate mechanical cues can facilitate cell cycle progression and accelerate division^17^^,^^18^; during metastasis and invasion, mechanical forces remodel the cytoskeleton and enhance migratory capacity, enabling tumor cells to breach tissue barriers and disseminate to distant sites.^19^^,^^20^ The pro-tumor effects of mechanical stimulation have been documented across various solid malignancies: in breast cancer, mechanotransductive signaling such as POSTN-INTEGRIN and YAP has been shown to mediate matrix stiffness-induced invasion^21^^,^^22^; in pancreatic cancer, targeting mechanical stress represents a promising strategy to enhance the efficacy of oxaliplatin^23^; in liver cancer, mechanical stress promotes thermotolerance via the SP1-IL4I1-AHR axis.^24^ Furthermore, mechanical stimulation influences tumor progression in lung^25^ and prostate^26^ cancers, among others, through distinct mechanisms. Notably, the colorectum is continuously subjected to diverse mechanical cues, including peristalsis and intraluminal pressure; however, matrix stiffness represents an experimentally tractable component of the mechanical microenvironment that may influence CRC development and progression, particularly metastasis. Investigating the relationship between mechanical stimulation and CRC may not only uncover unique pathogenic mechanisms and offer novel biomarkers for early detection but also open new avenues for therapeutic intervention, thereby significantly improving patient prognosis and highlighting both scientific and clinical relevance.

Based on this rationale, this study aims to integrate mechanosensitive and liver metastasis-associated genes to develop and validate a novel prognostic model for CRC using multiple machine learning approaches. The clinical utility of the model was systematically evaluated by analyzing differences in the immune microenvironment, drug sensitivity, and immunotherapy response between high- and low-risk patient groups stratified according to the model. Furthermore, quantitative reverse transcription PCR (qRT-PCR), western blot, and immunohistochemistry (IHC) were employed to examine the expression of the six model genes in normal colorectal epithelial cells versus CRC cell lines. Using a tunable substrate stiffness culture system, we validated the effect of matrix/substrate stiffness on the expression of these key genes in CRC cells *in vitro*. Finally, protein expression of SDC1 was assessed in CRC and adjacent normal tissues to further corroborate its clinical relevance. This study provides new theoretical and experimental foundations for prognostic assessment and targeted intervention in CRC from a mechanobiological perspective.

## Results

### Mechanosensitive gene set activity differs across CRC cell populations

To investigate the role of mechanosensitive genes in CRC, we compiled a gene set from a published transcriptomic dataset of DLD-1 colorectal adenocarcinoma cells cultured on soft substrates (2 and 55 kPa) versus collagen-coated glass coverslips (control). Compared to the control, 1,655 and 1,432 differentially expressed genes (DEGs) (|log_2_FC| ≥ 2) were identified on the 2 and 55 kPa substrates, respectively.^27^ The 323 genes that were consistently differentially expressed under both conditions (Table S3) were defined as mechanosensitive genes for subsequent single-cell analysis.

We obtained single-cell RNA sequencing data from three CRC patients with liver metastases. Batch effects across the samples were effectively removed using the Harmony package, resulting in a well-integrated dataset (Figure 1A). Following clustering analysis and annotation using established cell marker genes (Figures 1B and 1C), the 16 distinct clusters were classified as eight major cell types: tumor cells, T cells, NK cells, B cells, mast cells, myeloid cells, plasma cells, and cancer-associated fibroblasts.

**Figure 1:**
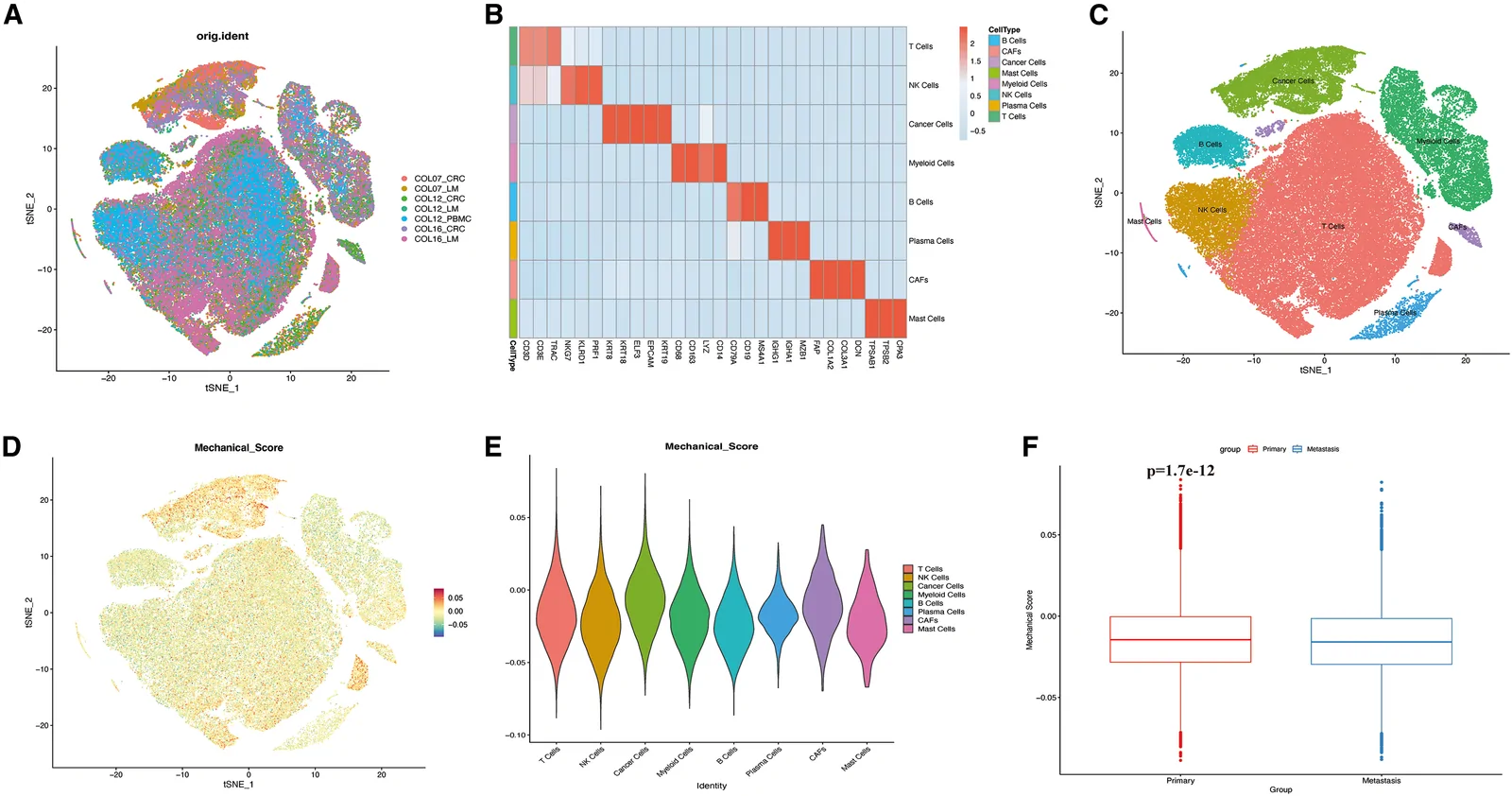
Characterization of the mechanosensitive gene set in single-cell transcriptomes (A) t-SNE plot showing cell clustering from integrated single-cell data. (B) Heatmap of expression for canonical cell marker genes used for annotation. (C) t-SNE plot visualizing the eight major cell types identified based on marker gene expression. (D) t-SNE plots displaying the distribution of individual cell marker genes. (E) Violin plot showing the descriptive distribution of mechanosensitive gene set activity scores across different cell types. No formal statistical comparison was performed for this figure. (F) Boxplot comparing the mechanosensitive scores between primary CRC tumors and matched liver metastases. Single-cell RNA-seq data were obtained from three CRC patients with matched primary tumor and liver metastasis samples (*n* = 3 patients). Statistical significance in (F) was determined by the paired Wilcoxon signed-rank test. The exact p value is shown in (F).

The activity of mechanosensitive signaling across these cell types was quantified using the “AddModuleScore” function in Seurat, based on the expression of the 323-gene set (Figure 1D). The analysis revealed that T cells and tumor cells exhibited higher mechanosensitive gene set scores, whereas mast cells and myeloid cells showed relatively lower scores (Figure 1E). Furthermore, primary tumor samples displayed significantly higher mechanosensitive scores than matched liver metastasis samples, although the magnitude of the difference was modest (Figure 1F).

Based on the median score, all cells were dichotomized into high and low mechanosensitivity groups. Differential expression analysis using the FindMarkers function identified 284 significant DEGs (false discovery rate [FDR] < 0.05, |log_2_FC| > 1) between these groups. This gene set was defined as the mechanosensitive gene signature in CRC tissue for subsequent analyses.

### Identification of CRC liver metastasis-associated genes

Liver metastasis is a critical factor contributing to poor prognosis and high mortality in CRC. To elucidate the specific mechanisms and intrinsic drivers of CRC liver metastasis, we integrated single-cell and bulk transcriptomic data, employing differential expression analysis and weighted gene co-expression network analysis (WGCNA) to identify liver metastasis-associated genes.

Differential expression analysis was performed between liver metastasis samples and primary tumor samples from the GSE41258, GSE49355, and GSE81558 datasets using the limma package, identifying DEGs based on thresholds of FDR < 0.05 and |log_2_FC| ≥ 1 (Figure 2A). In parallel, analysis of the single-cell data using the FindMarkers function identified 517 DEGs (FDR < 0.05, |log_2_FC| ≥ 1) between liver metastasis and primary tumor samples.

**Figure 2:**
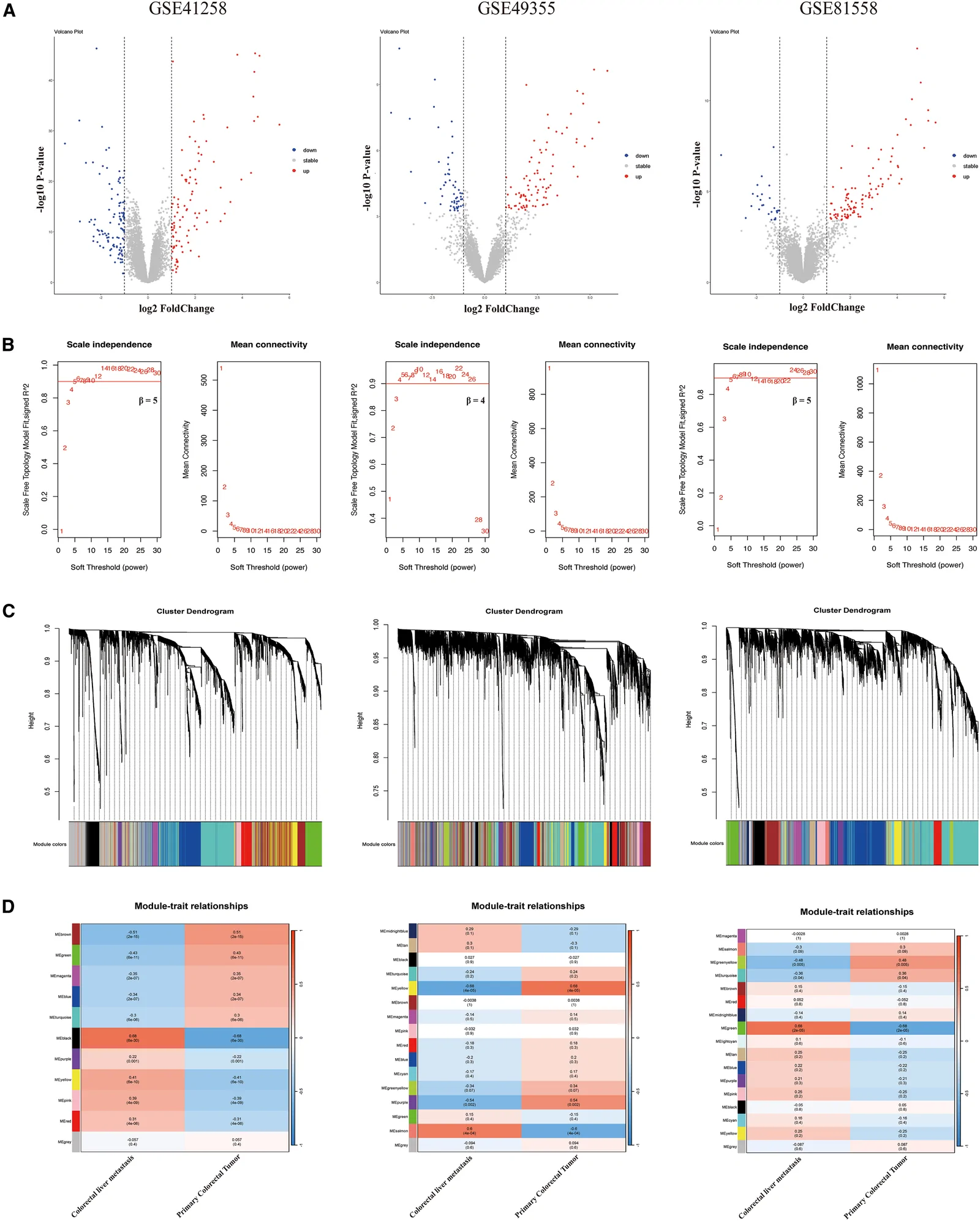
Identification of liver metastasis-associated genes in CRC (A) Volcano plots showing differentially expressed genes between primary tumors and liver metastases in the GSE41258, GSE49355, and GSE81558 datasets. (B) Scale-free topology analysis for selecting the soft-thresholding power in WGCNA for the three datasets. (C) Cluster dendrograms of genes based on the topological overlap matrix derived from the chosen soft threshold. (D) Heatmap of module-trait relationships showing the correlation between gene module eigengenes and the liver metastasis phenotype. Differential expression analysis in (A) was performed using the limma package with FDR < 0.05 and |log_2_FC| ≥ 1 as the significance thresholds. Module-trait correlations in (D) were assessed using Pearson correlation analysis, with correlation coefficients and *p* values shown in the heatmap.

To further delineate liver metastasis-associated genes, WGCNA was applied to the GSE41258, GSE49355, and GSE81558 datasets to identify co-expressed gene modules (Table S4). Soft thresholding powers were set to 5, 4, and 5 for the respective datasets (Figure 2B), resulting in signed R^2^ values greater than 0.85, indicating the attainment of approximate scale-free topology suitable for network analysis. Co-expression networks were constructed using the optimal soft thresholds, and genes were partitioned into distinct modules, with corresponding cluster dendrograms shown in Figure 2C. Module-trait relationships assessing correlation with the liver metastasis phenotype were calculated. The black module in GSE41258 (R = 0.68, *P* = 6e−30), the yellow module in GSE49355 (R = 0.68, *P* = 4e−05), and the green module in GSE81558 (R = 0.68, *P* = 2e−05) demonstrated the most significant correlations with liver metastasis (Figure 2D).

Genes from these significantly correlated modules were combined with the DEGs identified from both the bulk and single-cell analyses. This integrative approach yielded a final set of 1,708 genes associated with CRC liver metastasis.

### Construction of a mechanosensitive liver metastasis-associated prognostic signature

To identify genes jointly associated with mechanosensitivity and CRC liver metastasis, we intersected the mechanosensitive gene signature derived from high- versus low-scoring single-cell populations with the liver metastasis-associated gene set generated by integrating bulk transcriptomic differential expression analysis, single-cell differential analysis, and WGCNA. This step yielded 178 candidate genes implicated in both mechanosensitive responses and CRC liver metastasis (Figure 3A). Notably, the initial 323 mechanosensitive genes were used for single-cell module scoring and subsequent differential expression analysis, rather than being directly entered into the prognostic model. Univariate Cox regression analysis of these 178 candidate genes within the colorectal cancer cohort from The Cancer Genome Atlas (TCGA-CRC) identified 20 genes significantly associated with overall survival (Figure 3B).

**Figure 3:**
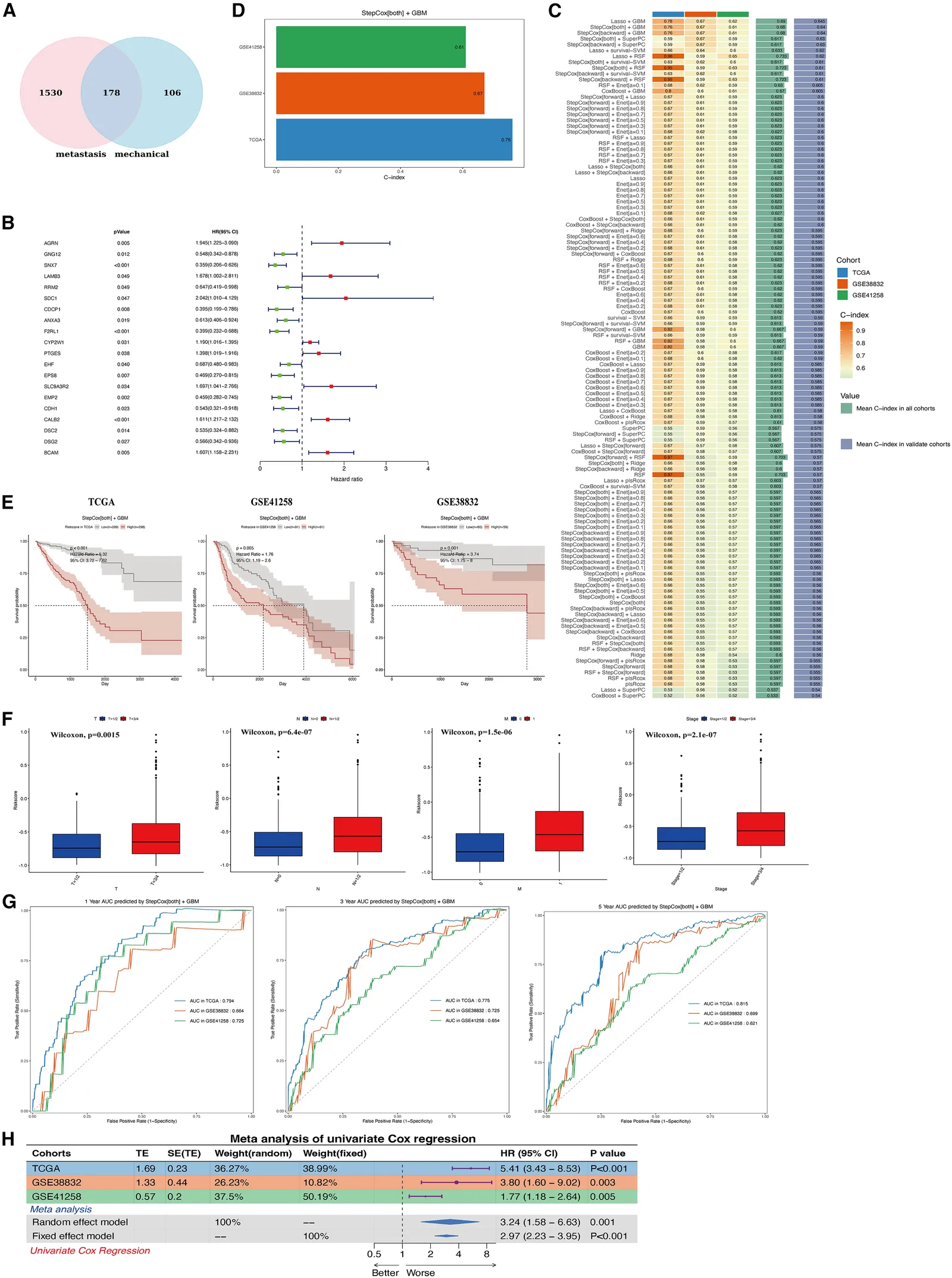
Construction and validation of the prognostic model (A) Venn diagram illustrating the intersection between the mechanosensitive gene set and liver metastasis-associated genes. (B) Forest plot of the univariate Cox regression analysis for the 20 prognostic genes identified from the intersection. (C) Bar plot of the C-indices for the 117 prediction models built by the Mime package on the test sets. (D) C-indices of the optimal StepCox[both]+GBM model in the training and test sets. (E) Kaplan-Meier survival curves for the high-risk and low-risk groups in the TCGA-CRC, GSE41258, and GSE38832 cohorts. (F) Boxplots showing the association between the risk score and tumor-node-metastasis (TNM) stage/American Joint Committee on Cancer (AJCC) stage in the TCGA-CRC cohort. (G) Time-dependent ROC curves analyzing the sensitivity and specificity for predicting 1-, 3-, and 5-year overall survival in the training and test sets. (H) Forest plot of the univariate Cox regression and meta-analysis for the StepCox[both]+GBM model across different cohorts. Kaplan-Meier survival curves in (E) were compared using the log rank test. Cox regression analysis was used for (B) and (H). Associations between risk score and clinicopathological stage in (F) were analyzed using the Wilcoxon rank-sum test or Kruskal-Wallis test, as appropriate. Exact *p* values are shown in the corresponding figures where applicable.

Subsequently, prognostic models were constructed based on these 20 genes using the Mime package, which integrated 10 machine learning algorithms and their combinations (totaling 117 models), across three independent CRC cohorts with survival data (one training set and two validation sets). Among all models, the Lasso+GBM combination achieved the highest average Concordance index (C-index = 0.69) across the training and validation sets. However, this model incorporated 10 feature genes (*AGRN*, *SNX7*, *SDC1*, *CDCP1*, *ANXA3*, *CYP2W1*, *PTGES*, *EPS8*, *EMP2*, and *CALB2*). In contrast, the StepCox[both]+GBM combination model, which utilized only six genes (*SNX7*, *SDC1*, *CDCP1*, *PTGES*, *EMP2*, and *CALB2*), achieved a comparable performance (C-index = 0.68). Considering the principles of model simplicity and clinical practicality, the StepCox[both]+GBM model was selected as the optimal prognostic signature (Figures 3C and 3D).

Based on the expression levels of these six genes and their respective risk coefficients, a risk score was calculated for each patient. Using the median risk score computed by the Mime package as the cutoff, CRC patients were stratified into high-risk and low-risk groups. The survival probability for each group was determined, and Kaplan-Meier analysis demonstrated that patients with higher risk scores had significantly poorer outcomes across all cohorts (Figure 3E). A quadrant diagram of TNM stage, AJCC stage, and risk scores in the TCGA-CRC cohort revealed that patients with higher risk scores had more advanced clinicopathological stages (Figure 3F).

### Model validation and evaluation

To further evaluate the prognostic value of the established model, ROC curves were plotted, and the AUC values at 1, 3, and 5 years were calculated for both the training and validation sets. In the TCGA-CRC cohort, the AUC values were 0.794, 0.775, and 0.815 for 1, 3, and 5 years, respectively. The corresponding AUC values were 0.664, 0.725, and 0.699 in the GSE38832 cohort and 0.725, 0.654, and 0.621 in the GSE41258 cohort (Figure 3G). These results indicate the model’s robust discriminative ability.

To determine the prognostic effect of the risk model, a meta-analysis of univariate Cox regression results was performed using the Mime package. The analysis demonstrated that the risk score was a significant prognostic factor for poorer overall survival in both the training set (TCGA-CRC: hazard ratio [HR] = 5.41, *p* < 0.001) and the validation sets (GSE38832: HR = 3.80, *p* = 0.003; GSE41258: HR = 1.77, *p* = 0.005) (Figure 3H).

A systematic literature review was conducted, identifying 74 previously published prognostic signatures for CRC (Table S5). These signatures were associated with various biological characteristics of CRC, including cell death, immunity, metabolism, and autophagy. The C-index of each of these 74 signatures was calculated across the different cohorts. Our model achieved the highest C-index in the TCGA-CRC and GSE41258 datasets and ranked second in the GSE38832 cohort (Figure S1). Univariate Cox regression analysis was performed for each model in all datasets to compare their association with survival outcomes. The results confirmed that our model was significantly associated with poorer survival in all cohorts (Figure S2). Similarly, compared to other risk signatures, our mechanosensitive risk signature achieved top-ranking AUC values at 1 year (Figure S3), 3 years (Figure S4), and 5 years (Figure S5) in the three independent cohorts. Collectively, these results support the prognostic utility of the liver metastasis-associated mechanosensitive risk signature in the available cohorts.

### Independent prognostic analysis

To assess whether the liver metastasis-associated mechanosensitive risk signature serves as an independent prognostic factor for CRC, univariate and multivariate Cox regression analyses were performed within the TCGA-CRC cohort. The univariate analysis demonstrated that the mechanosensitive risk score was significantly associated with overall survival (HR = 8.395, 95% confidence interval [CI]: 5.733–12.292, *p* < 0.001) (Figure 4A). After adjusting for other clinical variables in the multivariate analysis, the risk signature remained an independent prognostic factor (HR = 5.386, 95% CI: 3.435–8.445, *p* < 0.001) (Figure 4B).

**Figure 4:**
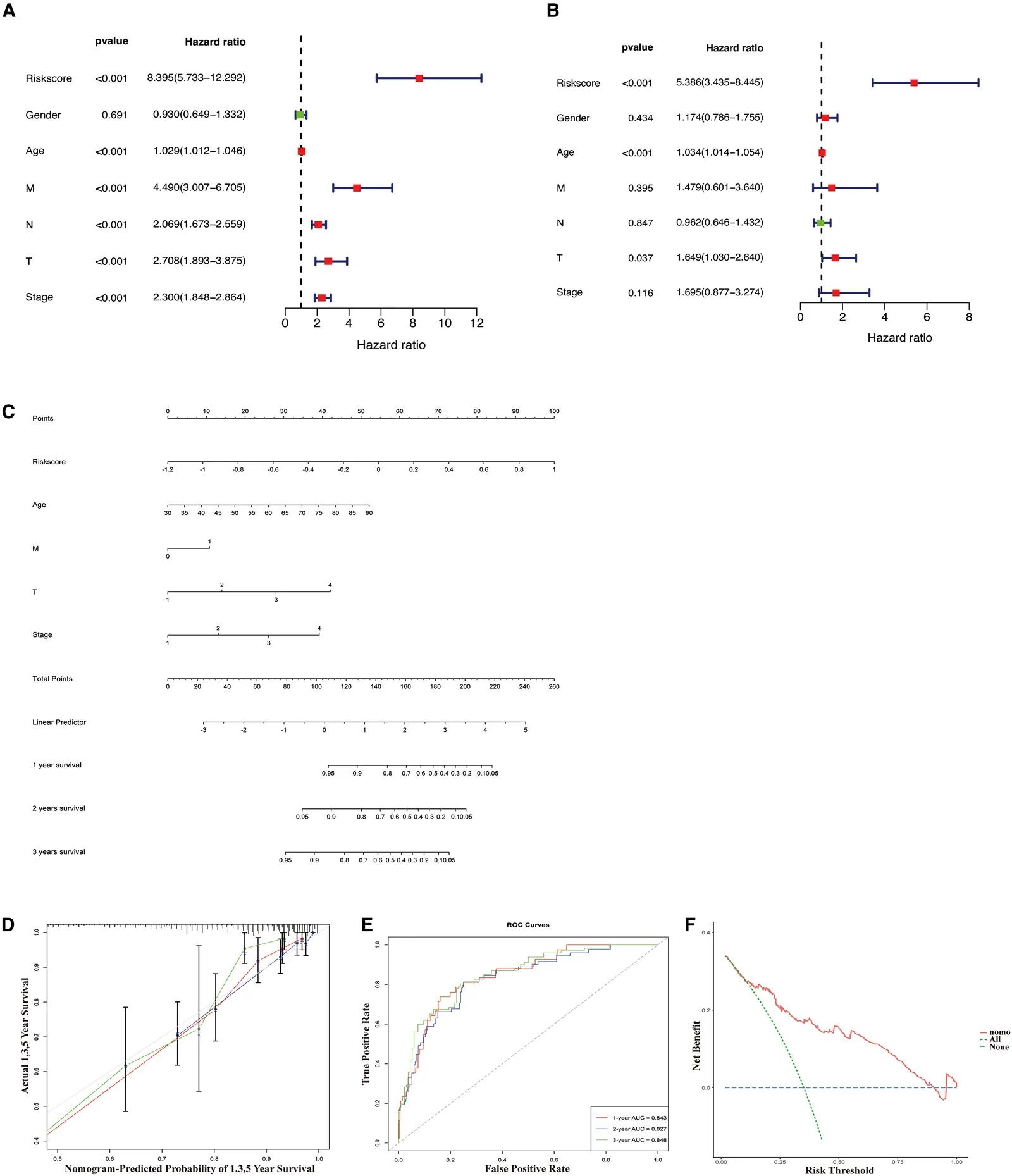
Independent prognostic analysis and nomogram construction (A) Forest plot of univariate Cox regression analysis of clinicopathological characteristics and the risk score in the TCGA-CRC cohort. (B) Forest plot of multivariate Cox regression analysis of clinicopathological characteristics and the risk score. (C) Prognostic nomogram integrating the risk signature and key clinical features for predicting 1-, 3-, and 5-year survival probability. (D) Calibration curves for the nomogram, comparing predicted versus observed survival probabilities. (E) ROC curves demonstrating the predictive performance of the nomogram at 1, 3, and 5 years. (F) DCA showing the net benefit of the nomogram compared to individual clinical features. Univariate and multivariate Cox proportional hazards regression analyses were used in (A) and (B), respectively. ROC and decision curve analyses were performed in the TCGA-CRC cohort. No additional hypothesis testing was performed for (C)–(F).

To enhance the clinical applicability of the risk signature, a nomogram was constructed by integrating the signature with key clinical characteristics from the TCGA-CRC cohort, providing a tool for individualized survival probability estimation at 1, 3, and 5 years (Figure 4C). The performance of the nomogram was evaluated by calibration curves, which showed excellent agreement between the predicted and observed survival probabilities (Figure 4D). The nomogram achieved high time-dependent AUC values of 0.843, 0.827, and 0.848 for predicting 1-, 3-, and 5-year survival, respectively (Figure 4E). Decision curve analysis (DCA) further confirmed the clinical utility of the nomogram, demonstrating a higher net benefit across a range of threshold probabilities compared to using clinical characteristics alone (Figure 4F). These findings indicate that the nomogram, based on the mechanosensitive risk signature, provides an accurate and reliable tool for personalized prognosis prediction in CRC patients.

### Potential molecular mechanisms underlying the mechanosensitive risk signature

To investigate the potential molecular mechanisms linking the risk signature to CRC patient prognosis, functional enrichment analysis was performed. Differential expression analysis between the high-risk and low-risk groups in the TCGA-CRC cohort using the limma package identified 2,229 DEGs (FDR < 0.05, |log_2_FC| > 0.25), comprising 869 upregulated and 1,360 downregulated genes.

GO enrichment analysis of the upregulated genes revealed significant enrichment in extracellular matrix (ECM)-related processes. The most prominent terms included “ECM organization” and “extracellular structure organization,” which contained the largest number of genes and exhibited highly significant adjusted *p* values. “Collagen-containing ECM” was also significantly enriched. Cellular components such as the “endoplasmic reticulum lumen” and “basement membrane,” as well as molecular functions including “integrin binding” and “collagen binding,” were enriched, albeit with slightly lower significance and fewer genes compared to the top terms (Figure S6A). KEGG pathway analysis indicated that the “cytoskeleton” pathway was the most significantly enriched, followed by the “PI3K-Akt signaling pathway” and “focal adhesion” (Figure S6B). These results suggest that biological processes related to the ECM and cytoskeleton pathways may play key roles in the high-risk group.

For the downregulated genes, GO analysis showed significant enrichment in biological processes such as “chromosome segregation,” “mitotic nuclear division,” and “organelle fission.” Enriched cellular components included the “mitochondrial matrix” and “chromosomal region,” linking them to energy metabolism and genetic material function, respectively. Molecular functions like “ligase activity” were prominent, indicating their importance in biosynthetic pathways, while “NADP binding” and “ribosomal binding” were also enriched, suggesting other potential mechanisms (Figure S6C). KEGG enrichment analysis highlighted pathways such as “cell cycle,” “cofactor biosynthesis,” and “peroxisome” with large numbers of genes and high statistical significance, pointing to their potential critical roles in cell proliferation and metabolic regulation. The enrichment of various metabolic pathways, including “fatty acid metabolism,” indicated active metabolic states, and the “peroxisome proliferator-activated receptor (PPAR) signaling pathway” may be involved in lipid metabolism and energy balance. Additionally, enrichment in pathways like “nucleotide sugar biosynthesis” and “nucleocytoplasmic transport” provides clues for understanding biosynthesis, cellular response, and repair mechanisms (Figure S6D). These findings illuminate the distinct molecular mechanisms that may underlie the different clinical outcomes observed between the risk groups defined by the mechanosensitive signature.

### Association between the risk signature and the tumor immune microenvironment

Tumor immune microenvironment heterogeneity is a critical factor influencing tumorigenesis, progression, and prognosis.^28^ To compare immune cell infiltration between the high-risk and low-risk groups, CIBERSORT analysis was employed. The results revealed that regulatory T cells and M0 macrophages were significantly elevated in the high-risk group, whereas resting dendritic cells, memory CD4^+^ T cells, and plasma cells were decreased (Figure 5A). A heatmap further illustrated the distinct immune cell infiltration patterns between the two groups (Figure 5B), suggesting that mechanical stimulation may be involved in the regulation of these immune populations.

**Figure 5:**
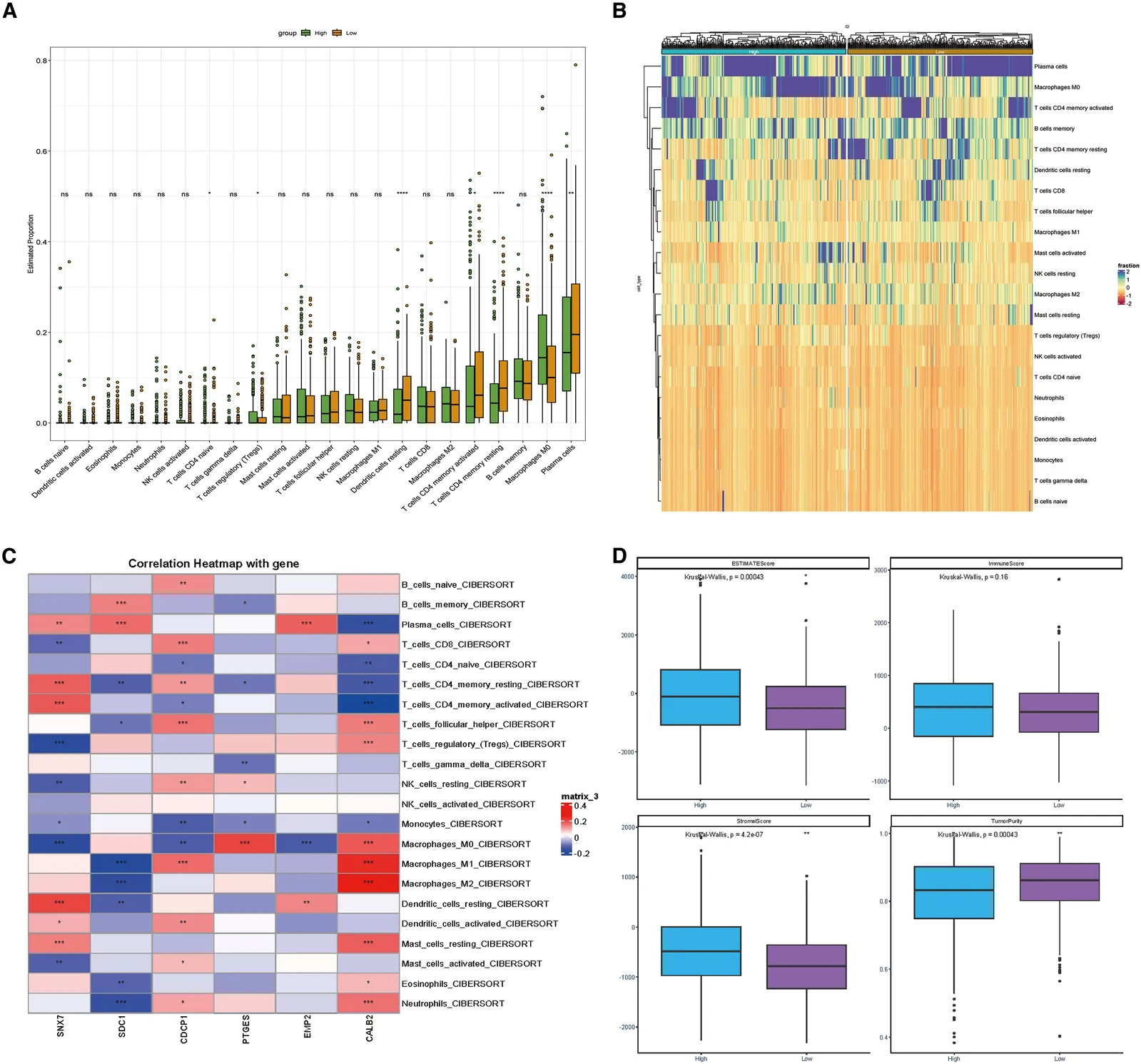
Immune infiltration analysis (A) Bar plot showing the differences in the infiltration levels of 22 immune cell types between the high-risk and low-risk groups. (B) Heatmap depicting the infiltration patterns of 22 immune cell types in the high-risk and low-risk groups. (C) Correlation heatmap between the six signature genes and the infiltration levels of various immune cells. (D) Boxplots comparing the stromal score, immune score, Estimation of Stromal and Immune cells in Malignant Tumor tissues using Expression data (ESTIMATE) score, and tumor purity between the risk groups. Differences between high-risk and low-risk groups in (A) and (D) were assessed using the Wilcoxon rank-sum test. Correlations in (C) were assessed using Spearman correlation analysis. Exact *p* values are shown in the corresponding figures where applicable. ns, not significant; ∗*p* < 0.05; ∗∗*p* < 0.01; ∗∗∗*p* < 0.001; and ∗∗∗∗*p* < 0.0001.

To further assess the correlation between the risk signature and immune cells, a correlation heatmap was generated for the six signature genes and various immune cell types (Figure 5C). The analysis indicated that *CALB2*, *PTGES*, *CDCP1*, and *SDC1* might be associated with macrophage polarization, *SNX7* potentially participates in the regulation of dendritic cells, and *EMP2* showed a certain correlation with plasma cells. The ESTIMATE algorithm was further applied to calculate the stromal score, immune score, ESTIMATE score, and tumor purity. The results demonstrated that the high-risk group exhibited significantly higher stromal and ESTIMATE scores, while the low-risk group had higher tumor purity (Figure 5D), indicating a more complex stromal and immune composition in the high-risk tumors.

### Association between the risk signature and somatic mutation patterns

To further explore the correlation between tumor mutation burden and the risk score, waterfall plots were used to visualize the somatic mutation landscape of individual CRC samples in the high-risk and low-risk groups. The top 15 most frequently mutated genes were largely consistent between the two subgroups; however, the overall mutation rate was higher in the high-risk group (96.28%) compared to the low-risk group (92.24%). *APC* and *TP53* were the two genes with the highest mutation frequencies in both groups. The mutation frequency of *KRAS* was higher in the low-risk group, whereas the high-risk group exhibited higher mutation frequencies in *MUC16*, *SYNE1*, and *FAT4*, along with a lower frequency of *KRAS* mutations. These results suggest a potential correlation between mechanical stimulation and the mutation patterns of these genes. Among all mutation types, missense mutations, nonsense mutations, and frameshift deletions were the three most predominant categories in both groups (Figures S7A and S7B).

### Association between the risk signature and predicted therapy response

The Tumor Immune Dysfunction and Exclusion (TIDE) online platform was utilized to predict the response to immunotherapy in the TCGA-CRC cohort. The analysis revealed that the high-risk group had significantly elevated TIDE scores (Figure 6A), exclusion scores (Figure 6B), and dysfunction scores (Figure 6C), alongside lower microsatellite instability (MSI) scores (Figure 6D). These exploratory results suggest that high-risk patients may have a greater potential for immune evasion and a poorer predicted response to immune checkpoint inhibitor therapy. A significant positive correlation was observed between the TIDE score and the risk score, further confirming that higher risk scores were associated with elevated TIDE scores (Figure 6E).

**Figure 6:**
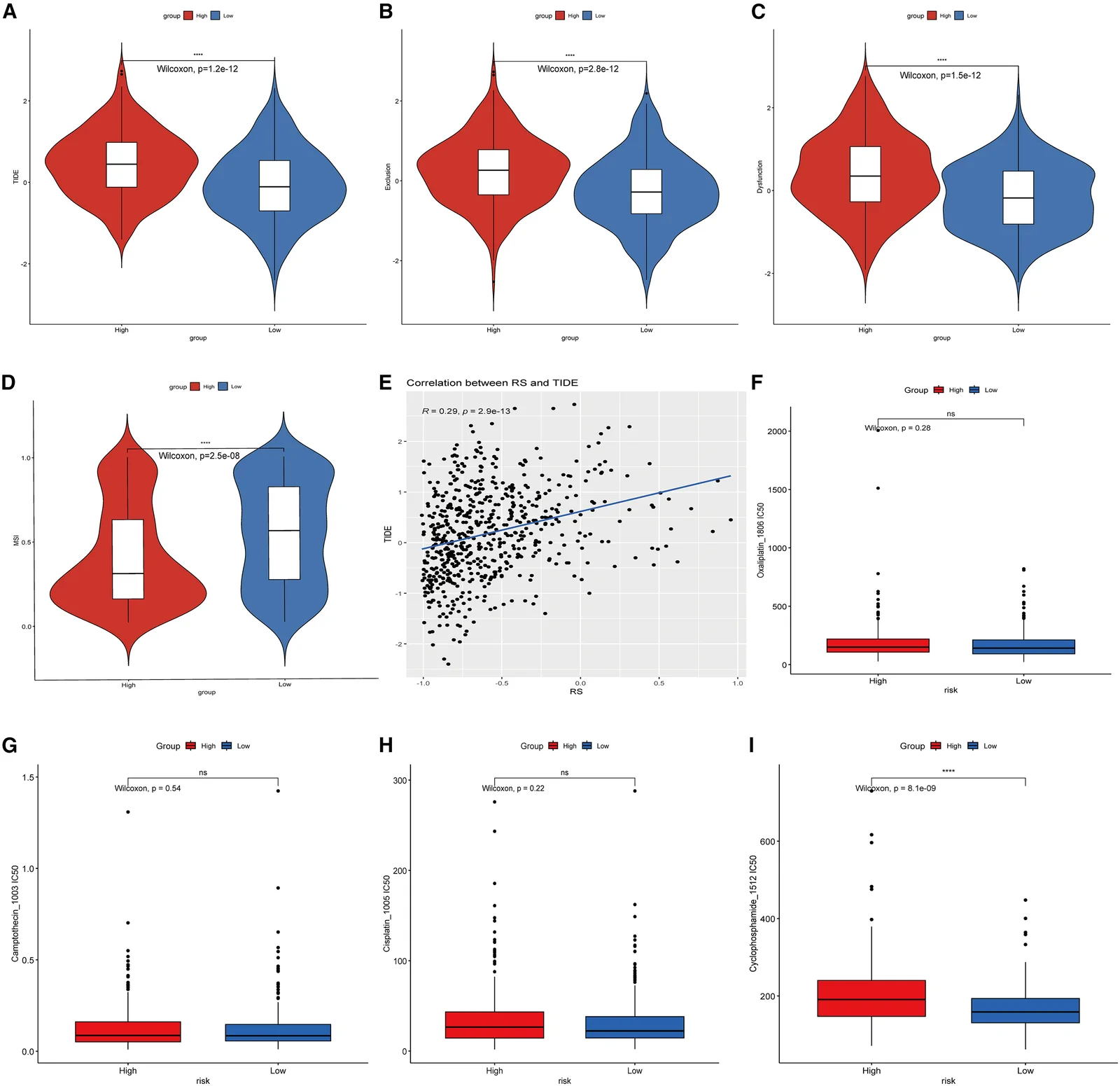
Analysis of immunotherapy response and drug sensitivity (A–D) Boxplots comparing the (A) TIDE score, (B) exclusion score, (C) dysfunction score, and (D) MSI score between the high-risk and low-risk groups in the TCGA-CRC cohort. (E) Scatterplot showing the correlation between the risk score and the TIDE score. (F–I) Boxplots comparing the estimated IC_50_ values for (F) oxaliplatin, (G) camptothecin, (H) cisplatin, and (I) cyclophosphamide between the risk groups. Boxplots show the median and interquartile range. Statistical significance in (A)–(D) and (F)–(I) was determined by the Wilcoxon rank-sum test. Correlation in (E) was assessed using Spearman correlation analysis. Exact *p* values are shown in the corresponding panels. ns, not significant; ∗*p* < 0.05; ∗∗*p* < 0.01; ∗∗∗*p* < 0.001; and ∗∗∗∗*p* < 0.0001.

Furthermore, the sensitivity to commonly used chemotherapeutic agents in CRC was assessed. No significant differences in the estimated IC_50_ values were observed between the high-risk and low-risk groups for oxaliplatin (Figure 6F), camptothecin (Figure 6G), or cisplatin (Figure 6H). In contrast, the IC_50_ value for cyclophosphamide was significantly higher in the high-risk group, suggesting a potential difference in predicted cyclophosphamide sensitivity between the two risk groups (Figure 6I).

### The six-gene signature shows cell-line-dependent responses to substrate stiffness

Based on prior analytical predictions, *CDCP1*, *SNX7*, and *EMP2* were classified as potential tumor suppressor genes, while *SDC1*, *PTGES*, and *CALB2* were predicted as potential oncogenes among the six identified risk signature genes. To validate these predictions and confirm their expression patterns in CRC, we quantified their mRNA levels in multiple CRC cell lines (RKO, DLD-1, HCT116, Caco-2, and HCT15) using the normal colonic epithelial cell line NCM460 as a control. The results demonstrated that the expression of *CDCP1*, *SDC1*, and *PTGES* was elevated in most CRC cell lines compared to NCM460. *CALB2* showed significantly increased expression only in RKO cells, while *SNX7* and *PTGES* were upregulated in both Caco-2 and HCT15 cells, with the most prominent increases observed in Caco-2 cells. *EMP2* was notably upregulated in Caco-2 cells (Figure 7A).

**Figure 7:**
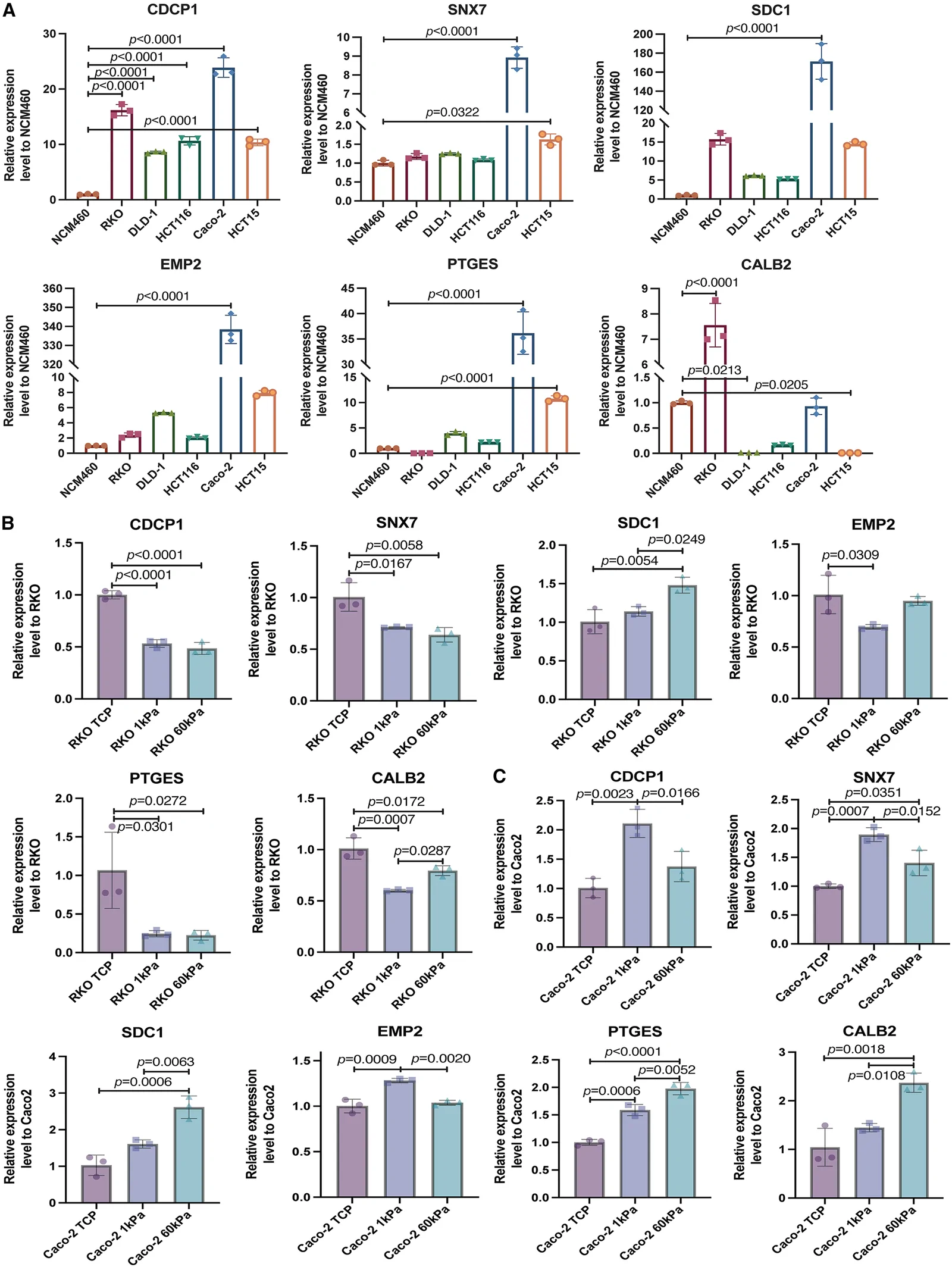
Validation of the six risk signature genes at the RNA level in cell lines and under substrate stiffness (A) qRT-PCR analysis of *CDCP1*, *SNX7*, *SDC1*, *EMP2*, *PTGES*, and *CALB2* mRNA expression in the normal epithelial cell line NCM460 and five CRC cell lines (RKO, DLD-1, HCT116, Caco-2, and HCT15). (B and C) qRT-PCR analysis of the six genes in (B) RKO and (C) Caco-2 cells cultured on tissue culture plastic (TCP), 1 kPa, or 60 kPa substrates for 48 h. Data are presented as mean ± SD from three independent experiments. Statistical significance was determined by one-way ANOVA followed by Dunnett’s multiple-comparison test for (A) and by one-way ANOVA followed by Tukey’s multiple-comparison test for (B) and (C). For qRT-PCR assays, technical triplicates were averaged for each biological replicate. Exact *p* values are shown in the corresponding figures.

To further investigate the response of these genes to mechanical stimulation, RKO and Caco-2 cells were cultured on standard TCP, as well as on soft (1 kPa) and stiff (60 kPa) hydrogel substrates. Analysis of gene expression revealed that substrate stiffness altered the expression levels of the six genes in a cell-line-dependent manner compared with the TCP control. Specifically, in RKO cells cultured on the stiffer (60 kPa) substrate, *CDCP1*, *SNX7*, *EMP2, PTGES*, and *CALB2* were downregulated, whereas *SDC1* was upregulated (Figure 7B). Similarly, in Caco-2 cells under high stiffness (60 kPa) conditions, *CDCP1*, *SNX7*, *EMP2*, *SDC1*, *PTGES*, and *CALB2* were upregulated (Figure 7C). These results indicate that the six signature genes exhibit stiffness-associated but cell-line-dependent expression patterns, rather than uniform responses across all CRC models. This heterogeneity may reflect differences in the intrinsic molecular background and mechanotransduction state of each cell line. Among the six genes, *SDC1* showed the most consistent stiffness-associated upregulation across the tested conditions, supporting its selection for further protein-level and functional validation.

### SDC1 is upregulated in CRC cells and tissues and responds to substrate stiffness

To further validate SDC1 expression at the protein level, we first examined SDC1 abundance in a panel of CRC cell lines and the normal colonic epithelial cell line NCM460. Western blot analysis showed that SDC1 protein expression was higher in several CRC cell lines than in NCM460, with relatively strong expression observed in Caco-2 cells and detectable expression in RKO, DLD-1, HCT116, and HCT15 cells (Figure 8A).

**Figure 8:**
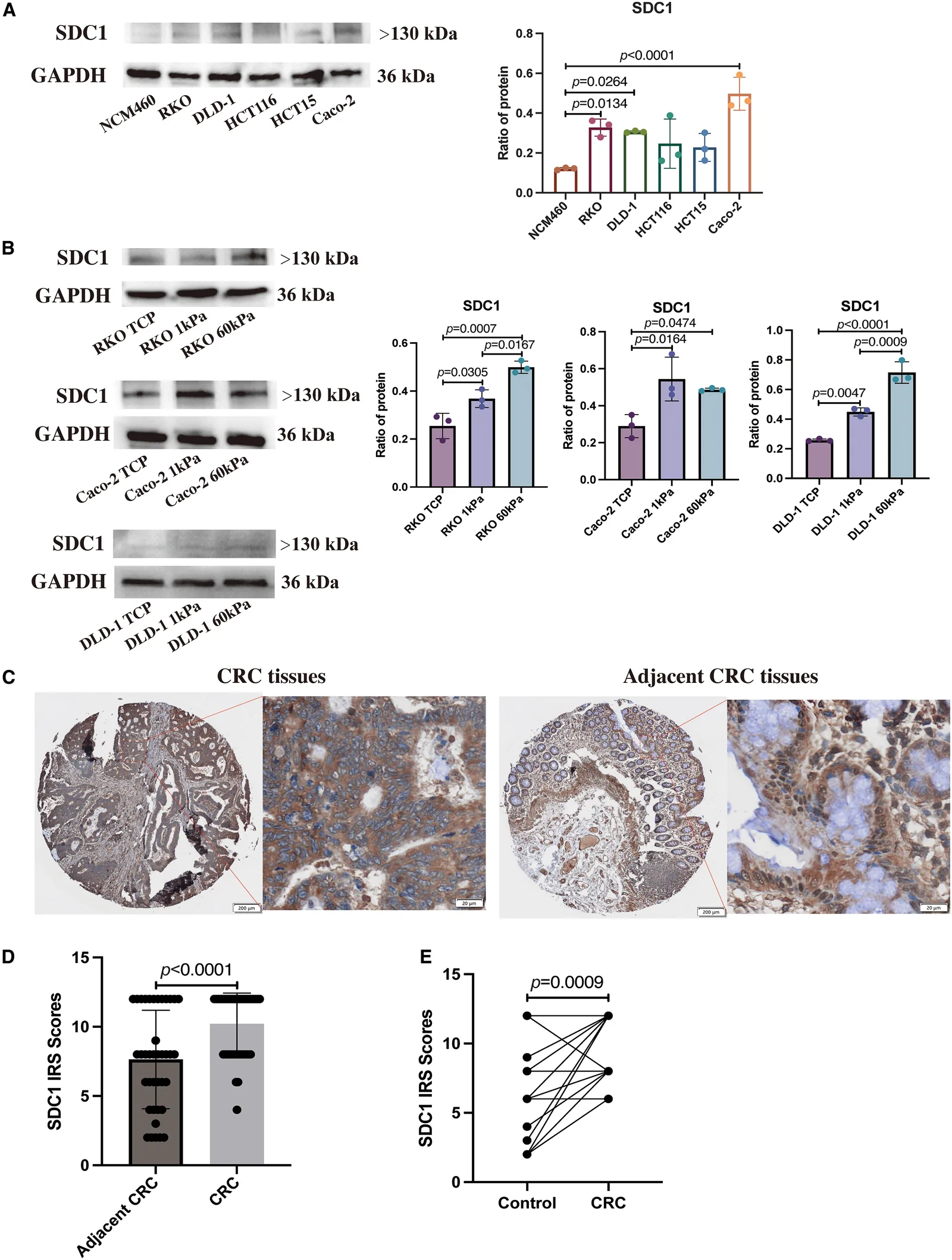
SDC1 protein expression in CRC cell lines, stiffness-modulated culture conditions, and CRC tissues (A) Western blot analysis of SDC1 protein expression in NCM460 and CRC cell lines, including RKO, DLD-1, HCT116, Caco-2, and HCT15. Cells were cultured on TCP under standard culture conditions. GAPDH was used as the loading control. (B) Western blot analysis and quantification of SDC1 expression in RKO, Caco-2, and DLD-1 cells cultured on TCP, 1 kPa, or 60 kPa substrates. GAPDH was used as the loading control. (C) Representative immunohistochemical staining images of SDC1 in CRC tissues and adjacent tissues, including representative low- and high-expression images and higher-magnification views. Scale bars, 200 μm in the left overview images and 20 μm in the right higher-magnification images. (D) Quantification of SDC1 IRS scores in CRC tissues (*n* = 75) and adjacent tissues (*n* = 39). IRS quantification was restricted to tumor-cell staining within the tumor region. (E) Paired comparison of SDC1 IRS scores between CRC tissues and matched adjacent tissues (*n* = 33 pairs). Quantified western blot data in (A) and (B) are presented as mean ± SD from three independent biological replicates. IRS data in (D) and (E) are presented as mean ± SD. Statistical significance was determined by one-way ANOVA followed by Dunnett’s multiple-comparison test for (A), one-way ANOVA followed by Tukey’s multiple-comparison test for (B), two-tailed Student’s *t-*test for (D), and paired two-tailed Student’s *t-*test for (E). Exact *p* values are shown in the corresponding panels. Full, uncropped western blot membranes corresponding to (A) and (B), including three independent experimental replicates, are provided in Figure S9.

We next assessed whether SDC1 protein expression was affected by matrix stiffness. RKO, Caco-2, and DLD-1 were selected because they represent distinct CRC molecular backgrounds. RKO is commonly characterized as an MSI/*BRAF*-mutant model, Caco-2 as an MSS/CIN-like epithelial model, and DLD-1 as an MSI/*KRAS*-mutant model. Published CMS annotations further suggest that these cell lines represent different transcriptional contexts, although CMS assignment in cell lines may vary depending on the classifier used.^29^^,^^30^ In RKO cells, SDC1 expression increased under 1 and 60 kPa conditions compared with TCP, with the highest expression observed under 60 kPa. In Caco-2 cells, SDC1 expression was also elevated under hydrogel culture conditions compared with TCP. Importantly, because the initial stiffness-associated gene set was derived from DLD-1 cells, we additionally examined SDC1 protein expression in DLD-1 cells cultured on TCP, 1 kPa, and 60 kPa substrates. SDC1 expression was increased in DLD-1 cells under stiffness-modulated culture conditions, particularly under 60 kPa, further supporting SDC1 as a stiffness-responsive candidate in CRC cells (Figure 8B).

We further evaluated SDC1 expression in CRC tissues by IHC using a tissue microarray. Representative staining showed stronger SDC1 expression in CRC tissues than in adjacent tissues (Figure 8C). Quantitative immunoreactive score (IRS) analysis confirmed that SDC1 expression was significantly higher in CRC tissues (*n* = 75) than in adjacent tissues (*n* = 39), and this difference was further confirmed in 33 paired CRC and adjacent tissue samples (Figures 8D and 8E).

### *SDC1* knockdown suppresses stiffness-associated migration and invasion in DLD-1 cells

To further determine whether *SDC1* contributes functionally to stiffness-associated CRC cell behavior, we performed *SDC1* knockdown experiments in DLD-1 cells. Among three shRNA candidates, shRNA1 and shRNA3 markedly reduced *SDC1* mRNA expression, with shRNA1 showing the strongest knockdown efficiency (Figure 9A). Therefore, shRNA1 was selected for subsequent functional assays. GFP fluorescence confirmed efficient transduction in both NC and shSDC1 DLD-1 cells, and bright-field imaging showed that *SDC1* knockdown was accompanied by evident morphological changes, with shSDC1 cells displaying a more rounded and clustered morphology compared with NC cells (Figure S8A). Consistently, western blot analysis further confirmed that SDC1 protein expression was substantially reduced in shSDC1 DLD-1 cells compared with NC cells (Figure 9B).

**Figure 9:**
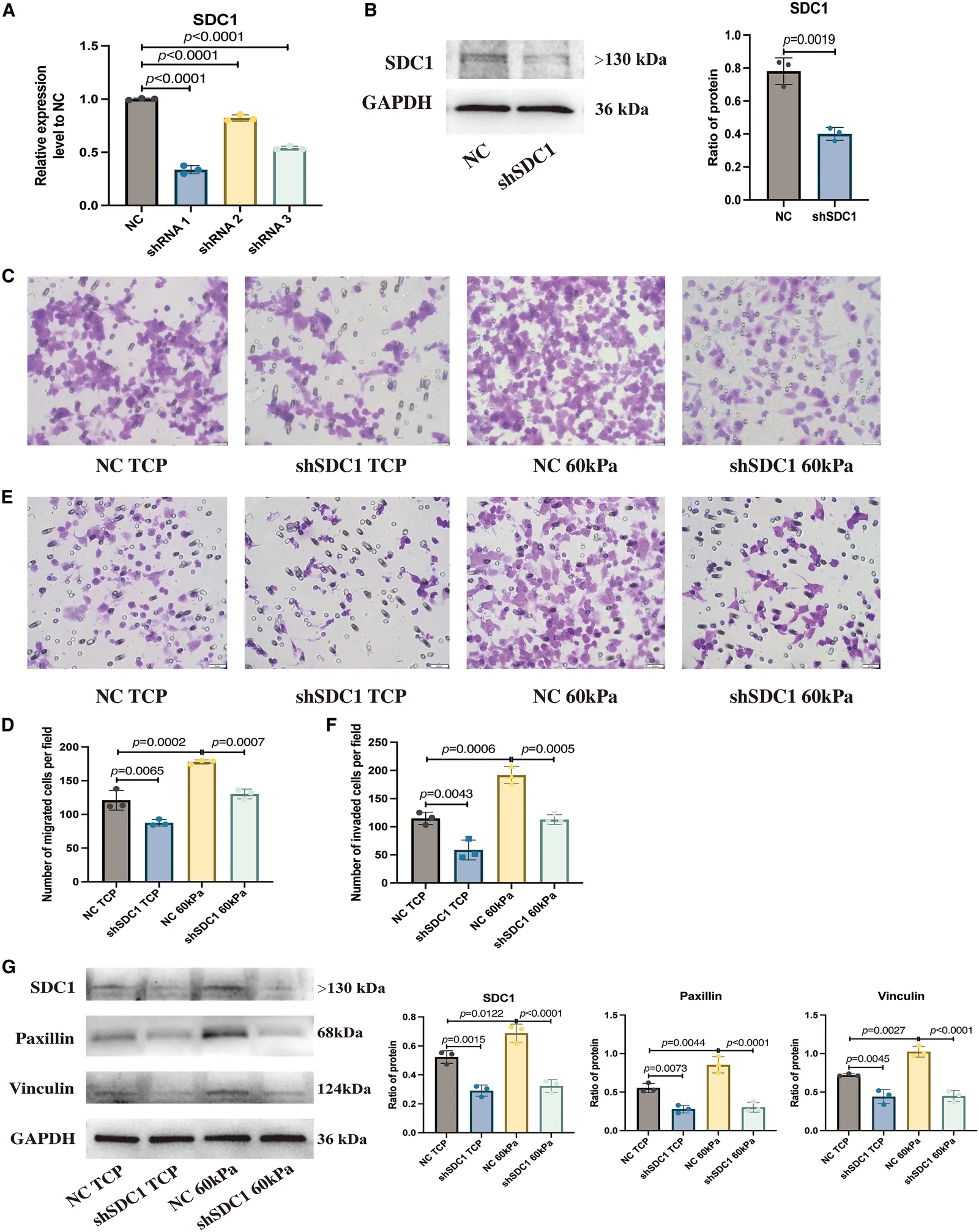
Functional validation of *SDC1* in DLD-1 cells under matrix stiffness conditions (A) qRT-PCR analysis of *SDC1* knockdown efficiency in DLD-1 cells transduced with three independent shRNAs targeting *SDC1*. NC cells served as the negative control. (B) Western blot analysis and quantification of SDC1 protein expression in NC and shSDC1 DLD-1 cells. GAPDH was used as the loading control. (C) Representative images of migrated DLD-1 cells under NC TCP, shSDC1 TCP, NC 60 kPa, and shSDC1 60 kPa conditions. (D) Quantification of migrated cells per field under the indicated conditions. (E) Representative images of invaded DLD-1 cells under NC TCP, shSDC1 TCP, NC 60 kPa, and shSDC1 60 kPa conditions. (F) Quantification of invaded cells per field under the indicated conditions. (G) Western blot analysis and quantification of SDC1, paxillin, and vinculin protein expression in DLD-1 cells under NC TCP, shSDC1 TCP, NC 60 kPa, and shSDC1 60 kPa conditions. GAPDH was used as the loading control. Scale bars, 20 μm in (C) and (E). Data are shown as mean ± SD from three independent experiments. For migration and invasion assays, each dot represents the average value of three randomly selected fields from one independent experiment. Statistical significance was determined by one-way ANOVA followed by Dunnett’s multiple-comparison test for (A), two-tailed Student’s *t* test for (B), and one-way ANOVA followed by Tukey’s multiple-comparison test for (D), (F), and (G). Full, uncropped western blot membranes corresponding to (B) and (G), including three independent experimental replicates, are provided in Figure S10. Exact *p* values are shown in the corresponding figures.

We next examined whether *SDC1* knockdown affected DLD-1 cell migration and invasion under different stiffness conditions. Under TCP and 60 kPa conditions, bright-field images further showed condition-dependent morphological differences among NC TCP, shSDC1 TCP, NC 60 kPa, and shSDC1 60 kPa cells, supporting that both matrix stiffness and *SDC1* knockdown were associated with altered DLD-1 cell morphology (Figure S8B). Under 60 kPa culture conditions, NC cells showed increased migratory and invasive capacities compared with cells cultured on TCP. In contrast, *SDC1* knockdown significantly reduced DLD-1 cell migration under both TCP and 60 kPa conditions (Figures 9C and 9D). Similarly, *SDC1* knockdown decreased the invasive capacity of DLD-1 cells, including under the 60 kPa condition (Figures 9E and 9F). These results suggest that *SDC1* contributes to the stiffness-associated migratory and invasive phenotype of DLD-1 cells.

Because substrate stiffness is closely linked to focal adhesion remodeling, we further assessed focal adhesion-associated proteins. Western blot analysis showed that 60 kPa culture increased the expression of SDC1, paxillin, and vinculin compared with TCP, whereas *SDC1* knockdown reduced the expression of these proteins under both TCP and 60 kPa conditions (Figure 9G). Together, these data support a functional association between SDC1, stiffness-enhanced migration/invasion, and focal adhesion-associated remodeling in CRC cells.

## Discussion

CRC ranks as the second most common malignancy in China, with its incidence steadily increasing. Distant metastasis is a primary contributor to poor prognosis in affected patients, ensuring that CRC will remain a significant threat to public health for the foreseeable future. Consequently, there is an urgent need to identify novel biomarkers for the early diagnosis and prognostic prediction of CRC. Recent research has highlighted the crucial role of the mechanical microenvironment in tumor initiation and progression. Given that the colorectum is perpetually subjected to various mechanical forces, the impact of biomechanics on its pathophysiology cannot be overlooked. However, the correlation between mechanosensitive genes and CRC prognosis remains largely unexplored. To our knowledge, this study is among the first to integrate a mechanosensitive signature with liver metastasis risk. The prognostic model we developed, based on six genes (*SNX7*, *SDC1*, *CDCP1*, *PTGES*, *EMP2*, and *CALB2*) identified from public databases, addresses this gap in knowledge. It provides a novel biomarker for individualized prognosis assessment in CRC and establishes a theoretical foundation for developing future therapeutic strategies that target the tumor’s mechanical microenvironment.

SNX7, a member of the sorting nexin (SNX) protein family, plays important roles in various cellular biological processes such as protein sorting, endocytosis, and endosomal signaling.^31^^,^^32^ Its aberrant expression has been significantly associated with tumor progression, therapy resistance, and poor prognosis in several cancers, including liver cancer, glioblastoma, and lung cancer.^33^^,^^34^^,^^35^ However, its role in CRC remains insufficiently studied. Our results indicate that higher *SNX7* expression may be associated with a favorable prognosis in CRC patients (HR = 0.359, *p* < 0.001). At the mRNA level, among the various CRC cell lines tested, only Caco-2 cells showed significantly elevated *SNX7* expression compared to the normal NCM460 cells, while no significant difference was observed in RKO, DLD-1, and HCT116 cells. Furthermore, substrate stiffness significantly altered *SNX7* expression in both RKO and Caco-2 cells cultured on 1 and 60 kPa hydrogels compared to standard TCP, although the specific expression trends differed between the two cell lines. These findings suggest a potential role for mechanical stiffness in regulating *SNX7* expression. Nevertheless, further experiments are required to elucidate how SNX7 responds to mechanical stimuli and its specific function in CRC liver metastasis.

CDCP1 is a transmembrane glycoprotein overexpressed in various solid tumors, including lung, breast, colorectal, and prostate cancers. It promotes cancer cell survival, metastasis, and therapy resistance by interacting with signaling molecules such as Src family kinases and PKCδ.^36^^,^^37^ Univariate Cox regression analysis in our study identified *CDCP1* as a potential protective factor for CRC patients (HR = 0.395, *p* = 0.008). However, qRT-PCR analysis revealed significantly elevated *CDCP1* mRNA expression in all five tested CRC cell lines compared to normal epithelial cells, a finding typically suggestive of an oncogenic role. Further analysis of the correlation between *CDCP1* and immune cell infiltration indicated a strong association with M0 and M1 macrophages, but not with M2 macrophages. This pattern suggests that CDCP1 might influence macrophage polarization toward the M1 phenotype. M1 macrophages are known to activate T cells and NK cells by secreting pro-inflammatory factors (e.g., TNF-α and IL-12), thereby enhancing anti-tumor immune responses and potentially contributing to better patient outcomes.^38^ Existing literature presents a complex picture of CDCP1’s function: its reduced expression was reported to promote metastasis in esophageal cancer,^39^ while in CRC, it has been shown to facilitate metastasis by regulating cell adhesion, migration, and invasion, with knockdown of *CDCP1* inhibiting the migratory and invasive capabilities of HCT116 cells.^40^ While current research predominantly focuses on the relationship between CDCP1 and tumor cells, its interaction with immune cells remains largely unexplored. Given its mechanosensitive properties identified in our study, future research should aim to elucidate CDCP1’s specific mechanisms within the tumor immune microenvironment and explore its potential as a prognostic biomarker or therapeutic target in CRC.

PTGES plays a significant role in tumorigenesis, progression, and prognosis, primarily through its regulation of prostaglandin E2 (PGE2) synthesis and associated downstream signaling pathways.^41^^,^^42^^,^^43^ In CRC, PTGES influences patient prognosis mainly by promoting chemotherapy resistance and facilitating immune escape. Studies have shown that elevated *PTGES* expression and PGE2 levels in CRC cells enhance glycolytic metabolism, leading to reduced sensitivity to 5-fluorouracil and consequently affecting patient outcomes.^44^ Furthermore, the PTGES/PGE2 axis contributes to an immunosuppressive microenvironment by recruiting tumor-associated immune cells and inhibiting T cell function, thereby promoting CRC progression.^45^ Our findings are consistent with existing literature, demonstrating that high *PTGES* expression is associated with reduced survival in CRC patients (HR = 1.398, *p* = 0.038). Regarding its responsiveness to mechanical stimulation, previous research in idiopathic pulmonary fibrosis has confirmed that matrix stiffness impairs the synthesis of anti-fibrotic lipid mediators, including prostaglandins, and reduces *PTGES* expression.^46^ In summary, PTGES may serve as a key effector molecule through which mechanical stimuli influence CRC development and progression.

EMP2, a cell surface protein, influences tumor progression in various cancers by modulating integrin signaling pathways.^47^ The role of EMP2 exhibits heterogeneity across different cancer types. For instance, in endometrial cancer and glioblastoma, high EMP2 expression is significantly associated with tumor aggressiveness, advanced stage, and poor prognosis.^47^ Conversely, in urothelial carcinoma, EMP2 has been identified as a tumor suppressor, with its low expression linked to disease progression.^48^ Our study suggests that *EMP2* may act as a protective factor in CRC patients (HR = 0.459, *p* = 0.002). However, research investigating the relationship between EMP2 and CRC remains limited. Furthermore, whether *EMP2* responds to mechanical stimulation and the specific mechanisms by which it might influence CRC pathogenesis require further experimental investigation.

CALB2, initially identified in the retina, is an intracellular calcium-buffering protein involved in various cellular functions and processes, including the modulation of intracellular calcium signaling, messenger targeting, cell-cycle arrest, and apoptosis.^49^ Current research indicates that CALB2 primarily influences CRC by regulating metastasis and chemosensitivity. Elevated CALB2 expression has been shown to enhance the invasive capacity of CRC cell lines DLD-1 and SW480.^50^ Conversely, it can also mediate 5-fluorouracil-induced apoptosis in CRC cells.^51^ The relationship between CALB2 and mechanical stimulation has not been directly reported. However, given its central role in regulating intracellular calcium signaling—a key mediator of mechanotransduction—it is plausible that CALB2 may interact with cellular contractility pathways. Given the dual role of CALB2 in potentially promoting metastasis while enhancing chemosensitivity in CRC, further experimental studies are warranted to clarify its precise function and the underlying mechanisms.

SDC1, also known as CD138, is a transmembrane heparan sulfate proteoglycan. Its core protein carries heparan sulfate and chondroitin sulfate glycosaminoglycan side chains and is primarily expressed on the basolateral surface of epithelial cells. Functioning as a cell adhesion molecule, SDC1 interacts with cell surface receptors and signaling molecules, participating in various physiological and pathological processes.^52^^,^^53^ SDC1 acts as a tumor-promoting factor in multiple cancers. In triple-negative breast cancer, high SDC1 expression inhibits the recruitment of tumor-infiltrating lymphocytes, leading to reduced patient survival.^54^ In pancreatic cancer, microRNA-494 can suppress tumor progression by downregulating *SDC1*,^55^ while serum SDC1 levels serve as a potential diagnostic biomarker in pancreatic ductal adenocarcinoma.^56^ However, findings regarding the role and clinical significance of SDC1 in CRC remain conflicting.^57^^,^^58^ Some studies indicate that knocking down *SDC1* reduces the invasive ability of HCT116 cells,^59^ which aligns with our findings suggesting its association with CRC liver metastasis. Nevertheless, reports on SDC1 expression levels in CRC versus adjacent tissues are inconsistent. One study involving 24 CRC and paired adjacent tissues demonstrated significantly higher mRNA and protein expression of SDC1 in CRC.^60^ Conversely, another study comprising 477 CRC and 65 adjacent tissues found lower SDC1 protein levels in tumor tissues, with loss of SDC1 expression indicating poor prognosis.^58^ To clarify this discrepancy, we examined SDC1 protein expression in 75 CRC and 39 adjacent tissues. Our results showed significantly elevated SDC1 protein in CRC tissues, a finding confirmed in 33 paired CRC and adjacent tissue samples. Together with univariate Cox regression analysis identifying *SDC1* as a prognostic risk factor in CRC (HR = 2.042, *p* = 0.047), its elevated expression in CRC cell lines compared with NCM460, and IHC validation, these findings support an association between SDC1 expression and increased prognostic risk in CRC. Regarding its mechanosensitive properties, SDC1 binds ECM components (e.g., laminin) via its extracellular domain, regulating integrin-mediated focal adhesion formation.^61^ It mediates EMT through the Wnt/β-catenin pathway, and its nuclear translocation can suppress the mesenchymal phenotype.^62^ Under mechanical stress such as stretching or fluid shear stress, SDC1 may coordinate the switch between collective migration and single-cell invasion by regulating E-cadherin/N-cadherin conversion. In the present study, *SDC1* knockdown in DLD-1 cells reduced migration and invasion under both TCP and 60 kPa conditions and altered focal adhesion-associated proteins, supporting a functional association between SDC1, stiffness-enhanced malignant behavior, and focal adhesion remodeling in CRC cells.

Our study extends beyond prognostic signature development by suggesting a potential mechanobiological program associated with CRC progression. The six-gene model suggests that the mechanical forces inherent to the colorectum are not merely passive bystanders but active drivers of a transcriptional program favoring metastasis, particularly to the liver. While *SDC1* emerged as the most consistent stiffness-responsive candidate in our validation experiments, the other five signature genes showed more heterogeneous responses across cell lines and stiffness conditions. This suggests that the six-gene signature should be interpreted primarily as a prognostic and metastasis-associated transcriptional model, rather than as a uniformly stiffness-regulated gene module. The heterogeneity among these genes may reflect cell-line-specific molecular backgrounds, differentiation states, oncogenic alterations, and intrinsic mechanotransduction programs. Future research should prioritize delineating the context-specific roles of these genes in mechanotransduction and validating their therapeutic relevance. Targeting key components of this mechanosensitive program, perhaps in combination with conventional therapies, could offer a novel strategy to disrupt the metastatic cascade in CRC.

### Limitations of the study

Several limitations should be acknowledged. First, the mechanosensitive gene set was derived from a single *in vitro* study using DLD-1 cells, which may not fully capture the complexity of mechanosensitive responses *in vivo*. In addition, the single-cell analysis was based on three paired CRC samples, which further limits the breadth of inference. The relatively lower mechanosensitive score observed in CAFs does not exclude their well-established role in ECM production and remodeling, but likely reflects the tumor-cell-centered origin of the DLD-1-derived stiffness-responsive gene set used in this study. Although primary tumors showed a slightly higher mechanosensitive score than liver metastases, this transcriptional score should not be interpreted as a direct measurement of tissue stiffness. Previous atomic force microscopy measurements have shown that CRC liver metastases can be stiffer than primary colorectal tumors.^63^ This apparent discrepancy may reflect differences between tissue-level mechanical properties and the expression of mechanosensing-related genes, which can also be influenced by cellular composition, tumor purity, tumor-cell state, and site-specific transcriptional adaptation. Therefore, the current findings should be interpreted as hypothesis-generating and warrant validation in larger independent datasets. Second, while our six-gene signature showed prognostic performance in public cohorts, prospective validation in larger, multi-center clinical cohorts is warranted. Because external assessment cohorts were considered during final model selection, additional fully independent prospective validation with prespecified model parameters will be needed to further confirm generalizability. Moreover, this signature was designed for prognostic risk stratification and does not define a specific step of the metastatic cascade, such as invasion, intravasation, extravasation, or metastatic colonization. Although comparisons between primary tumors from patients with and without metastasis may further clarify metastatic potential, this analysis was beyond the primary aim of the present prognostic modeling study. In addition, the TIDE and oncoPredict analyses were exploratory and require validation in independent clinical cohorts or experimental treatment-response models before therapeutic conclusions can be drawn. Hepatocyte co-culture systems or liver-mimetic three-dimensional models will be needed to further evaluate the roles of *SDC1* and other signature genes in the liver metastatic microenvironment. Third, the functional mechanisms underlying SDC1-mediated mechanotransduction in CRC liver metastasis remain to be fully elucidated; future studies should investigate downstream pathways such as YAP/TAZ and integrin signaling. Finally, the stiffness-tunable hydrogel experiments were conducted in two CRC cell lines, and further validation in additional cell lines and three-dimensional culture models would strengthen the findings.

## Resource availability

### Lead contact

Further information and requests for resources and reagents should be directed to and will be fulfilled by the lead contact, Ziran Xu (xuziran@jlu.edu.cn).

### Materials availability

This study did not generate new unique reagents.

### Data and code availability

• Transcriptomic and clinical data from TCGA-COAD and TCGA-READ are available at https://portal.gdc.cancer.gov/.
• GEO datasets (GSE38832, GSE41258, GSE81558, GSE49355, and GSE178318) are available at https://www.ncbi.nlm.nih.gov/geo/.
• Tissue microarray data are available from Shanghai Outdo Biotech Company upon reasonable request with permission.
• Core analysis code supporting the main bioinformatics and machine-learning analyses has been deposited at GitHub and is publicly available at https://github.com/crc-ml-research/CRC-prognostic-machine-learning.
• Any additional information required to reanalyze the data reported in this paper is available from the lead contact upon request.

## Acknowledgments

This study was supported by the 10.13039/501100001809National Natural Science Foundation of China (grant no. 82401608), the 10.13039/100007847Natural Science Foundation of Jilin Province (YDZJ202501ZYTS137) and the 10.13039/501100004032Jilin University Baiqiu’en Program (grant no. 2025B34).

## Author contributions

T.Y. and C.L. conceived and designed the study; T.Y. performed the bioinformatics analyses, machine learning modeling, and data curation; Y.Y. and X.L. performed validation experiments and investigations; J.W. and C.L. provided resources and contributed to data visualization; Z.X. supervised the study, administered the project, and acquired funding; T.Y. drafted the manuscript, and Z.X. revised the manuscript. All authors participated in the preparation of figures and the manuscript, and all authors read and approved the final manuscript.

## Declaration of interests

The authors declare no competing interests.

## STAR★Methods

### Key resources table

**Table:** 

REAGENT or RESOURCE	SOURCE	IDENTIFIER
**Antibodies**		

SDC1 rabbit monoclonal antibody	HUABIO	Cat# ET1703-42; RRID: AB_3070399
Paxillin antibody [N7H16]	Selleck Chemicals	Cat# F1106; RRID: AB_3756884
Vinculin antibody [M20M17]	Selleck Chemicals	Cat# F0110; RRID: AB_3756883
GAPDH mouse monoclonal antibody	Engibody	Cat# E3Z0901
HRP-conjugated anti-rabbit IgG	Yeasen Biotechnology	Cat# 771526ES60
HRP-conjugated anti-mouse IgG	Yeasen Biotechnology	Cat# 771475ES60

**Biological samples**		

Human colorectal cancer tissue microarray	Shanghai Outdo Biotech Company	OD-CT-DgCol02-003
Human colorectal cancer tissue microarray	Shanghai Outdo Biotech Company	HCol-Ade060CS-01
Human colorectal cancer tissue microarray	Shanghai Outdo Biotech Company	HRec-Ade060PG-01

**Chemicals, peptides, and recombinant proteins**		

RPMI-1640 medium	BBI	Cat# E600028
Fetal bovine serum	BBI	Cat# E510008
Penicillin-streptomycin	BBI	Cat# E607011
TRIzol reagent	BBI	Cat# B610409
RIPA lysis buffer	Beyotime	Cat# P0013B
PMSF protease inhibitor	Beyotime	Cat# ST507
BCA protein assay kit	Beyotime	Cat# P0012
ECL detection reagents	Yeasen Biotechnology	Cat# 36208ES60

**Critical commercial assays**		

Hifair® II cDNA Synthesis Kit	Yeasen Biotechnology	Cat# 11121ES60
Hieff™ SYBR Green Master Mix	Yeasen Biotechnology	Cat# 11201ES08

**Deposited data**		

TCGA colon adenocarcinoma transcriptomic and clinical data	The Cancer Genome Atlas	TCGA-COAD; https://portal.gdc.cancer.gov/
TCGA rectal adenocarcinoma transcriptomic and clinical data	The Cancer Genome Atlas	TCGA-READ; https://portal.gdc.cancer.gov/
Colorectal cancer expression dataset	Gene Expression Omnibus	GSE38832
Colorectal cancer expression dataset	Gene Expression Omnibus	GSE41258
Colorectal cancer liver metastasis expression dataset	Gene Expression Omnibus	GSE81558
Colorectal cancer liver metastasis expression dataset	Gene Expression Omnibus	GSE49355
Colorectal cancer single-cell RNA-seq dataset	Gene Expression Omnibus	GSE178318
Core bioinformatics and machine-learning analysis code	This paper	https://github.com/crc-ml-research/CRC-prognostic-machine-learning

**Experimental models: Cell Lines**		

Human NCM460 normal colon mucosal epithelial cells	HySigen Biosciences Inc.	RRID: CVCL_0460
Human RKO colorectal cancer cells	HySigen Biosciences Inc.	RRID: CVCL_0504
Human DLD-1 colorectal cancer cells	HySigen Biosciences Inc.	RRID: CVCL_0248
Human HCT116 colorectal cancer cells	HySigen Biosciences Inc.	RRID: CVCL_0291
Human Caco-2 colorectal cancer cells	HySigen Biosciences Inc.	RRID: CVCL_0025
Human HCT-15 colorectal cancer cells	HySigen Biosciences Inc.	RRID: CVCL_0292

**Oligonucleotides**		

*SNX7* qRT-PCR primers	This paper	Sequences provided in Table S1
*SDC1* qRT-PCR primers	This paper	Sequences provided in Table S1
*CDCP1* qRT-PCR primers	This paper	Sequences provided in Table S1
*PTGES* qRT-PCR primers	This paper	Sequences provided in Table S1
*EMP2* qRT-PCR primers	This paper	Sequences provided in Table S1
*CALB2* qRT-PCR primers	This paper	Sequences provided in Table S1
*GAPDH* qRT-PCR primers	This paper	Sequences provided in Table S1
Negative-control shRNA	This paper	5′-GTTCTCCGAACGTGTCACGTT-3′
*SDC1* shRNA-1	This paper	5′-GACTGCTTTGGACCTAAAT-3′
*SDC1*shRNA-2	This paper	5′-GAGCAGGACTTCACCTTTGAA-3′
*SDC1* shRNA-3	This paper	5′-CCGCAAATTGTGGCTACTAAT-3′

**Recombinant DNA**		

Negative-control shRNA plasmid	PPL	Target sequence provided in Table S2
*SDC1*shRNA plasmids	PPL	Target sequences provided in Table S2

**Software and algorithms**		

R, version 4.1.0	R Foundation for Statistical Computing	https://www.r-project.org/
sva	Bioconductor	https://bioconductor.org/packages/sva/
Seurat	Satija Lab	https://satijalab.org/seurat/
Harmony	GitHub	https://github.com/immunogenomics/harmony
WGCNA	CRAN	https://cran.r-project.org/package=WGCNA
Mime	GitHub	https://github.com/l-magnificence/Mime
limma	Bioconductor	https://bioconductor.org/packages/limma/
survival	CRAN	https://cran.r-project.org/package=survival
survminer	CRAN	https://cran.r-project.org/package=survminer
timeROC	CRAN	https://cran.r-project.org/package=timeROC
rms	CRAN	https://cran.r-project.org/package=rms
ggDCA	CRAN	https://cran.r-project.org/package=ggDCA
CIBERSORT	Stanford University	https://cibersort.stanford.edu/
ESTIMATE	MD Anderson Cancer Center	https://bioinformatics.mdanderson.org/estimate/
ggpubr	CRAN	https://cran.r-project.org/package=ggpubr
corrr	CRAN	https://cran.r-project.org/package=corrr
pheatmap	CRAN	https://cran.r-project.org/package=pheatmap
TIDE	Dana-Farber Cancer Institute	http://tide.dfci.harvard.edu/
oncoPredict	GitHub	https://github.com/dingj35/oncoPredict
GraphPad Prism 8	GraphPad Software	https://www.graphpad.com/
ImageJ	National Institutes of Health	https://imagej.nih.gov/ij/

**Other**		

Softwell® polyacrylamide hydrogel plates, 1 kPa and 60 kPa	Flexcell cellsoft	Softwell® plates

### Experimental model and study participant details

#### Cell lines and culture

The human normal colon mucosal epithelial cell line NCM460 (RRID:CVCL_0460) and CRC cell lines RKO (RRID:CVCL_0504), DLD-1 (RRID:CVCL_0248), HCT116 (RRID:CVCL_0291), Caco-2 (RRID:CVCL_0025), and HCT15 (RRID:CVCL_0292) were kindly provided by HySigen Biosciences Inc. Supplier-provided quality-control reports included STR profiling and PCR-based mycoplasma testing, and all cell lines were reported to be mycoplasma-negative. All cell lines were cultured in RPMI-1640 medium (BBI) supplemented with 10% fetal bovine serum and 1% penicillin-streptomycin at 37 °C in a 5% CO_2_ humidified atmosphere.

Cells were routinely passaged using 0.25% trypsin-EDTA upon reaching 90% confluence. For cryopreservation, cells were resuspended in freezing medium containing 10% DMSO and stored in liquid nitrogen after gradient freezing.

#### Culture on stiffness-tunable substrates

Cells were seeded at a density of 5 × 10^5^ cells per 6-cm dish onto standard tissue culture plastic (TCP) or polyacrylamide hydrogels with calibrated stiffnesses of 1 kPa and 60 kPa (e.g., Softwell® plates, Matrigen). Cells were allowed to adhere and grow for 48 hours prior to any subsequent experiments.

#### Human tissue microarray samples

The study involving human colorectal cancer (CRC) tissues was conducted in accordance with the Declaration of Helsinki and was approved by the Ethics Committee of Shanghai Outdo Biotech Company (approval numbers: SHYJS-CP-1407018, SHYJS-CP-1410002, and SHYJS-CP-1304026). All patients provided written informed consent for participation. Available clinical and demographic details of the tissue microarray samples are provided in Table S6.

### Method details

#### Data acquisition and preprocessing

Transcriptomic data and corresponding clinical information for the training set were obtained from The Cancer Genome Atlas (TCGA) cohorts for colon adenocarcinoma (TCGA-COAD) and rectal adenocarcinoma (TCGA-READ) via the GDC portal (portal.gdc.cancer.gov). To mitigate potential batch effects arising from technical variations between the two cohorts, the ComBat algorithm (implemented in the R sva package), based on a negative binomial generalized linear model, was applied to adjust the gene expression profiles.

Normal tissue samples were excluded from subsequent analysis. Quality control was performed on the raw data by filtering out low-abundance genes that exhibited zero expression in more than 30% of the samples. Additionally, samples with incomplete survival information or an overall survival time of less than 30 days were removed. The resulting curated dataset was used for subsequent model training and analysis.

For external validation, dataset GSE38832 and GSE41258 were downloaded from the Gene Expression Omnibus (GEO) database (https://www.ncbi.nlm.nih.gov/gds/), along with their respective platform annotation files. The raw expression matrices for each dataset underwent background correction, quantile normalization, and probe summarization. Similarly, samples lacking complete survival information or with an overall survival time of less than 30 days were excluded, forming the independent test sets for further validation.

#### Single-cell RNA sequencing data processing

Single-cell RNA sequencing data from primary tumor and matched liver metastasis samples of three CRC patients were obtained from the GSE178318 dataset. Data analysis was performed using the Seurat package in R. Quality control was applied to filter high-confidence cells based on the following thresholds, determined by visual inspection of data distributions (violin plots): (1) percentage of mitochondrial gene counts (percent.mt)<25% to exclude low-quality or dying cells, and (2) number of unique genes detected per cell (nFeature_RNA)<7,000 to remove outliers potentially resulting from technical artifacts. The Harmony package was employed to integrate the datasets and remove batch effects across samples. Cell clusters were identified using the “FindNeighbors” and “FindClusters” functions and visualized using t-SNE projection. Cell types were annotated based on canonical marker genes.

A mechanosensitive gene set was compiled through literature curation. The raw count matrix was extracted from the Seurat object using the “GetAssayData” function, and the intersection between the detected genes and the predefined mechanosensitive gene set was identified. The “AddModuleScore” function in Seurat was used to calculate an activity score for the mechanosensitive gene set in each cell. Cells were then dichotomized into high and low mechanosensitive groups based on the median score. Differentially expressed genes (DEGs) between these two groups were systematically identified for subsequent analyses.

#### Identification of mechanosensitive genes associated with CRC liver metastasis

Gene expression datasets related to CRC liver metastasis (GSE81558, GSE41258, GSE49355) were downloaded from the GEO database, including their raw expression matrices and corresponding platform annotation files. Normal colon and adjacent non-tumor tissue samples were systematically excluded based on predefined criteria.

Weighted Gene Co-expression Network Analysis (WGCNA) was performed using the “WGCNA” R package. A suitable soft-thresholding power was first determined to ensure a scale-free network topology. Based on this, a signed adjacency matrix was constructed and subsequently transformed into a topological overlap matrix (TOM). Gene co-expression modules were identified through average linkage hierarchical clustering of the TOM-based dissimilarity measure. The gene module demonstrating the highest correlation with the liver metastasis phenotype was selected for downstream analysis.

To comprehensively identify liver metastasis-associated genes, we employed a dual approach: (1) DEGs between primary tumor and liver metastasis samples were identified from the bulk transcriptomic datasets using the limma R package; (2) The FindMarkers function in Seurat was applied to the single-cell RNA sequencing data (GSE178318) to identify DEGs between primary and metastatic samples at the single-cell resolution.

The mechanosensitive gene set derived from single-cell analysis was then intersected with the liver metastasis-associated genes identified through the above analyses. This integrative approach yielded 178 candidate mechanosensitive genes potentially involved in CRC liver metastasis, which were used for subsequent prognostic model construction.

#### Prognostic model construction using integrative machine learning

A comprehensive machine-learning framework was implemented using the Mime R package. A total of 117 individual models and algorithm combinations were constructed from 10 machine-learning approaches, including random survival forest, elastic net, least absolute shrinkage and selection operator (Lasso) Cox regression, ridge regression, stepwise Cox regression, CoxBoost, partial least squares regression for Cox models (plsRcox), supervised principal component analysis (SuperPC), generalized boosted regression models (GBM), and survival support vector machines (survival-SVMs).

Gene expression data from the TCGA-CRC, GSE38832, and GSE41258 cohorts were normalized separately before model analysis. TCGA-CRC was designated as the model-development cohort. Univariate Cox regression was performed exclusively in the TCGA-CRC cohort for the 178 candidate genes, and 20 genes significantly associated with overall survival at *p* < 0.05 were retained as candidate predictors for subsequent machine-learning analysis.

All 117 candidate models were trained exclusively using the TCGA-CRC cohort. Feature selection, parameter estimation, model fitting, and hyperparameter tuning were conducted using TCGA-CRC data, with internal cross-validation applied where required by the corresponding algorithms. After model training, the fitted models were applied directly to GSE38832 and GSE41258 without additional feature selection, model refitting, or hyperparameter adjustment.

Model discrimination was assessed using the concordance index in the TCGA-CRC cohort and the two external assessment cohorts. The final model was selected by considering both prognostic discrimination and the consistency of performance across the three cohorts. Models showing high apparent performance in TCGA-CRC but poor performance in the external cohorts were considered potentially overfitted. The StepCox[both]+GBM model was ultimately selected because it demonstrated comparatively stable and reproducible prognostic performance across multiple cohorts.

For the final model, bidirectional stepwise Cox regression was first used to select the predictors entered into the GBM model. The GBM was fitted using a Cox proportional hazards loss function, with a maximum of 10,000 candidate trees, an interaction depth of 3, a minimum of 10 observations per terminal node, and a learning rate of 0.001. Ten-fold cross-validation within the TCGA-CRC cohort was used to determine the optimal number of boosting trees. Patient-specific risk scores were calculated from the linear-predictor output of the fitted Cox-GBM model, with higher scores indicating a higher predicted mortality risk.

#### Model evaluation and validation

Based on the median risk score derived from the optimal model, patients in both the training and validation sets were stratified into high-risk and low-risk groups. The Kaplan-Meier survival analysis was performed using the “survminer” R package to assess the model’s ability to distinguish patient outcomes between these groups. The time-dependent receiver operating characteristic (ROC) curves were generated with the “timeROC” package to evaluate the model’s predictive accuracy at 1, 3, and 5 years.

To benchmark the performance of our model against existing prognostic signatures, we systematically retrieved previously published CRC risk models from the PubMed database. The prognostic efficacy of these published models was assessed by calculating their C-index and time-dependent area under the curve (AUC) values at 1, 3, and 5 years within our training and validation cohorts. Comparative analysis was conducted to evaluate the relative performance of our optimal model against these established signatures.

#### Construction and validation of a prognostic nomogram

Clinical and pathological data from the TCGA-CRC cohort were collected, including survival status, survival time, age, TNM stage, overall stage, and risk score. Univariate Cox proportional hazards regression analysis was first performed using the “survival” R package to identify statistically significant prognostic variables. Variables with significant associations in the univariate analysis were subsequently incorporated into a multivariate Cox regression model to establish an integrated prognostic assessment system.

To enhance clinical applicability, a prognostic nomogram was constructed using the “rms” R package, integrating all independent prognostic factors identified from the multivariate analysis. The nomogram was designed to predict 1-, 3-, and 5-year overall survival probabilities. Calibration curves were plotted to evaluate the agreement between predicted and observed survival outcomes. Additionally, decision curve analysis (DCA) was performed using the “ggDCA” package to assess the clinical utility of the nomogram by quantifying the net benefit across different threshold probabilities over time.

#### Functional enrichment analysis

DEGs between the high-risk and low-risk groups, as defined by the optimal prognostic model, were identified using the “limma” R package. Genes meeting the thresholds of false discovery rate (FDR)<0.05 and absolute log_2_ fold change (|log_2_FC|)>0.25 were considered significant. To elucidate the underlying biological mechanisms, the upregulated and downregulated gene sets were analyzed separately.

Gene Ontology (GO) enrichment analysis was performed to characterize the DEGs across three functional categories: biological processes, molecular functions, and cellular components. Additionally, Kyoto Encyclopedia of Genes and Genomes (KEGG) pathway analysis was conducted to identify significantly enriched signaling pathways. These analyses collectively provided a comprehensive functional profile of the risk-associated gene signatures.

#### Tumor microenvironment analysis

To investigate the relationship between the mechanosensitive liver metastasis risk signature and immune cell infiltration, the CIBERSORT algorithm was employed to quantify the relative abundances of 22 immune cell types within each sample. Differences in immune cell infiltration between the high-risk and low-risk groups were visualized using the “ggpubr” R package. Correlations between the six risk signature genes and the infiltrating immune cells were calculated using the correlate function from the “corrr” package. Results were presented as a correlation heatmap generated with the “pheatmap” package, with a statistical significance threshold set at *p* < 0.05. Furthermore, the “ESTIMATE” algorithm was applied to calculate stromal, immune, and tumor purity scores for each sample, enabling the characterization of the overall tumor microenvironment. Differences in these scores between the risk groups were compared and visualized using box plots.

#### Analysis of immunotherapy response and drug sensitivity

The Tumor Immune Dysfunction and Exclusion (TIDE) score was utilized to evaluate the potential for immune evasion and predict response to immune checkpoint inhibitors. Normalized gene expression data from the TCGA-CRC cohort were submitted to the TIDE web platform (http://tide.dfci.harvard.edu/) to calculate individual TIDE scores. Higher TIDE scores indicate a greater likelihood of immune escape and a poorer predicted response to immunotherapy. Differences in TIDE scores between the high-risk and low-risk groups were compared and visualized using boxplots.

Drug sensitivity was assessed using data from the Genomics of Drug Sensitivity in Cancer database. The “oncoPredict” R package was employed to estimate the half-maximal inhibitory concentration (IC50) of various chemotherapeutic agents for patients in each risk group. This method correlates tumor gene expression profiles with known drug response patterns from cancer cell lines. Differences in predicted IC50 values between the high-risk and low-risk groups were statistically evaluated using the Wilcoxon rank-sum test, with results presented using box plots.

#### qRT-PCR

Gene expression of the six risk-feature genes was analyzed by qRT-PCR using SYBR Green chemistry. Total RNA was extracted with Trizol reagent and reverse transcribed using the Hifair® II cDNA Synthesis Kit (Yeasen Biotechnology). Quantitative PCR was performed using HieffTM SYBR Green Master Mix (Yeasen Biotechnology) on a QuantStudio® system. Reactions were run in triplicate under standard cycling conditions. Primer sequences are listed in Table S1. Relative expression was calculated using the 2ˆ^(−ΔΔCt)^ method with *GAPDH* as the reference gene.

#### Western blot analysis

Protein expression of SDC1 was analyzed by western blot. Cells were lysed in RIPA buffer containing PMSF protease inhibitor on ice for 30 min, followed by centrifugation at 12,500 rpm for 20 min at 4 °C. Protein concentration was determined using a BCA assay kit (Beyotime). Equal amounts of protein (30 μg per lane) were separated by SDS-PAGE and transferred to PVDF membranes. After blocking with 5% non-fat milk for 1 h at room temperature, membranes were incubated overnight at 4 °C with primary antibodies against SDC1 (1:2000; HUABIO), paxillin (1:1000; selleck), vinculin (1:10,000; selleck) and GAPDH (1:10,000; Engibody). Following TBST washes, membranes were incubated with HRP-conjugated secondary antibodies for 1 h at room temperature. Protein bands were visualized using ECL detection reagents.

#### IHC

Tissue microarray sections were baked overnight at 70 °C, deparaffinized in xylene, and rehydrated through a graded ethanol series. Antigen retrieval was performed using citrate buffer under high pressure. Endogenous peroxidase activity was quenched with 3% H_2_O_2_. After blocking with serum, sections were incubated overnight at 4 °C with a primary antibody against SDC1 (1:100 dilution). The following day, sections were washed and incubated with an HRP-conjugated secondary antibody at 37 °C for 40 min. Signal was developed using DAB, and nuclei were counterstained with hematoxylin. Sections were then dehydrated, cleared, and mounted with neutral resin. Stained slides were scanned using an Olympus VS200 whole-slide scanner. Each tissue microarray core represented one independent patient sample. No duplicate cores from the same patient were included; therefore, each core was treated as one biological sample for IHC quantification. Immunoreactivity was independently evaluated by two pathologists blinded to patient data using the Immunoreactive Score (IRS), calculated as staining intensity score multiplied by the percentage of positive cells score. Discrepant scores were reviewed jointly, and a consensus score was used for subsequent analysis. SDC1 staining was mainly observed in the membrane and cytoplasm of tumor cells, with occasional stromal staining; however, IRS quantification was restricted to tumor-cell staining within the tumor region. IRS scores of 0-4 were defined as low expression, and IRS scores of 5-12 were defined as high expression.

#### *SDC1* shRNA transfection in DLD-1 cells

DLD-1 cells were transfected with shRNA plasmids targeting *SDC1* or a negative control shRNA. The shRNA target sequences are listed in Table S2. Cells were transfected when they reached approximately 40-50% confluence. Before transfection, the culture medium was removed, and cells were washed once or twice with PBS, followed by one wash with RPMI-1640 medium. The medium was then replaced with antibiotic-free complete medium. For each well, 2 μg of plasmid DNA was diluted in 200 μL Opti-MEM® I medium and gently mixed. Subsequently, 6 μL X-tremeGENE HP DNA Transfection Reagent was added to the diluted plasmid solution, mixed gently, and incubated at room temperature for 15-30 min to allow complex formation. The transfection mixture was then added dropwise to each well, and the culture plate was gently rotated to ensure even distribution. GFP fluorescence was monitored to assess transfection efficiency. After selection of NC and shRNA-1-transfected cells with 4 μg/mL puromycin, the percentage of GFP-positive cells exceeded 95%, and these cells were used for subsequent experiments. Cells were harvested 72 h after transfection for RNA extraction, protein extraction, and subsequent functional assays.

#### Transwell migration and invasion assays

Transwell migration and invasion assays were performed using 24-well Transwell chambers. For invasion assays, Matrigel, pipette tips, microcentrifuge tubes, and Transwell inserts were pre-cooled at 4 °C before use. Matrigel was diluted 1:8 in serum-free medium, and 60 μL of diluted Matrigel was carefully added to the upper surface of each Transwell insert without introducing bubbles. The inserts were incubated at 37 °C for 30 min to 1 h to allow Matrigel polymerization. Before cell seeding, 50 μL serum-free medium was added to the upper chamber to hydrate and equilibrate the Matrigel for 30 min. For migration assays, uncoated Transwell inserts were used.

NC and shSDC1 DLD-1 cells were cultured on TCP or 60 kPa substrates for 48 h. Cells were then collected and resuspended in serum-free medium at a concentration of 5×10ˆ5 cells/mL. Complete medium containing 10% FBS was added to the lower chamber of each well, and 100 μL of cell suspension, corresponding to 5×10ˆ4 cells, was added to the upper chamber. Care was taken to avoid bubbles. Cells were incubated under standard culture conditions for 24 h.

After incubation, the Transwell inserts were removed, and the medium was discarded. The inserts were washed twice with PBS for 5-10 min each time and fixed with 4% paraformaldehyde for 20-30 min. After fixation, the inserts were washed twice with PBS for 5-10 min each time and stained with 0.1% crystal violet for 15-20 min. Non-migrated or non-invaded cells on the upper surface of the membrane were gently removed using cotton swabs moistened with PBS. The inserts were then washed twice with PBS for 5-10 min each time. Migrated or invaded cells on the lower surface of the membrane were imaged under a microscope at 400×magnification. For each independent experiment, three randomly selected fields were imaged and quantified using ImageJ.

### Quantification and statistical analysis

All quantitative experimental data were derived from three independent biological replicates unless otherwise stated and are presented as the mean ± standard deviation (SD). For qRT-PCR assays, technical triplicates were averaged before statistical analysis. Statistical analyses were performed using GraphPad Prism 8 software. For qRT-PCR data, relative gene expression was calculated using the 2ˆ^(−ΔΔCt)^ method, with *GAPDH* used as the reference gene for normalization.

For western blot analysis, band intensities were quantified using ImageJ. The intensity of each target protein band was normalized to the corresponding GAPDH band from the same sample. Relative protein expression was then calculated by normalizing to the indicated control group. For comparisons between two groups, an unpaired two-tailed Student’s *t*-test was used. For comparisons among three or more groups, one-way ANOVA followed by Dunnett’s or Tukey’s multiple-comparison test was used, as appropriate. Dunnett’s multiple-comparison test was used when multiple groups were compared with a single control group, whereas Tukey’s multiple-comparison test was used for pairwise comparisons among multiple groups.

For migration and invasion assays, migrated or invaded cells were imaged under a microscope at 400×magnification. For each independent experiment, three randomly selected fields were quantified, and the average value was used as one biological replicate. Quantification was performed using ImageJ. Differences among NC TCP, shSDC1 TCP, NC 60kPa, and shSDC1 60kPa groups were analyzed using one-way ANOVA followed by Tukey’s multiple-comparison test.

Differences between CRC tissues and adjacent tissues were assessed using Student’s *t*-test. For paired CRC and adjacent tissue samples, paired two-tailed Student’s *t*-test was used. One-way ANOVA followed by Dunnett’s multiple-comparison test was used to compare SDC1 protein expression among CRC cell lines and the NCM460 cell line. One-way ANOVA followed by Tukey’s multiple-comparison test was used to analyze expression differences under different substrate stiffness conditions, including TCP, 1 kPa, and 60 kPa, in RKO, Caco-2, and DLD-1 cells. A *p* value of less than 0.05 was considered statistically significant. Exact *p* values are shown in the corresponding figure panels where applicable. *p* values are reported to four decimal places, with values smaller than 0.0001 reported as *p* < 0.0001.

## Supplemental information

Document S1. Figures S1–S10 and Tables S1–S4, related to Figures 1–3, 7, 8, and 9

Table S5. The 74 previously published prognostic signatures, related to Figure 3

Table S6. Clinical and demographic details of tissue microarray samples, related to Figure 8
