# Supplementary material for: Integrative genomics and experimental validation identify a mechanosensitive gene signature associated with colorectal cancer liver metastasis
Source: iScience. 2026 Oct 16;29(11):117926. doi: 10.1016/j.isci.2026.117926 (PMC13696149; doi:10.1016/j.isci.2026.117926)
Supplement: Supplementary file 1 [file mmc1.pdf]

iScience, Volume *29*

Supplemental information

Integrative genomics and experimental validation identify a mechanosensitive gene signature associated with colorectal cancer liver metastasis

Tianxue Yang, Ye Yuan, Xue Li, Jiaqi Wang, Chenyao Li, and Ziran Xu

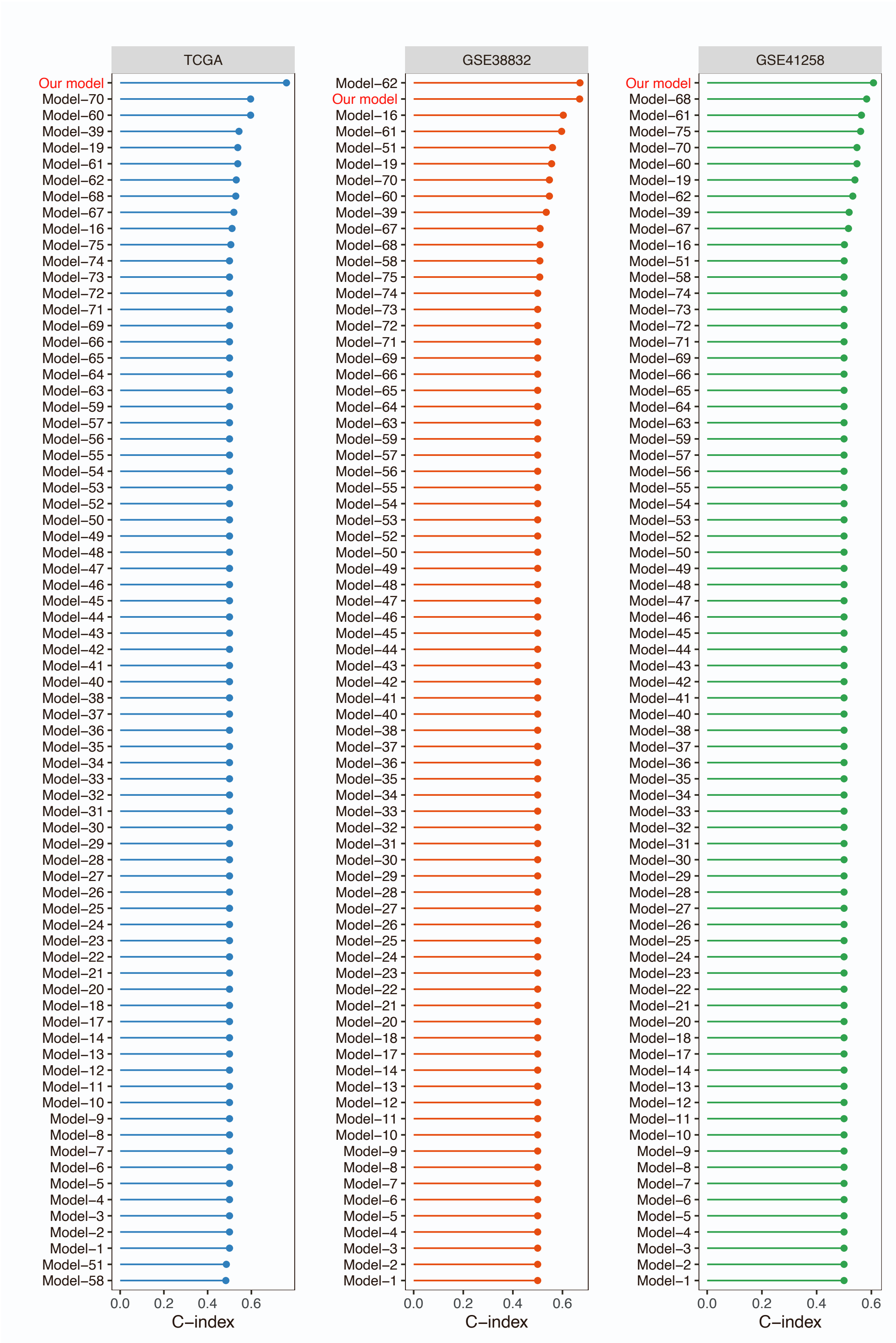

Figure S1. Comparison of the C-index between the StepCox[both]+GBM model and 74 previously published prognostic models, related to Figure 3.

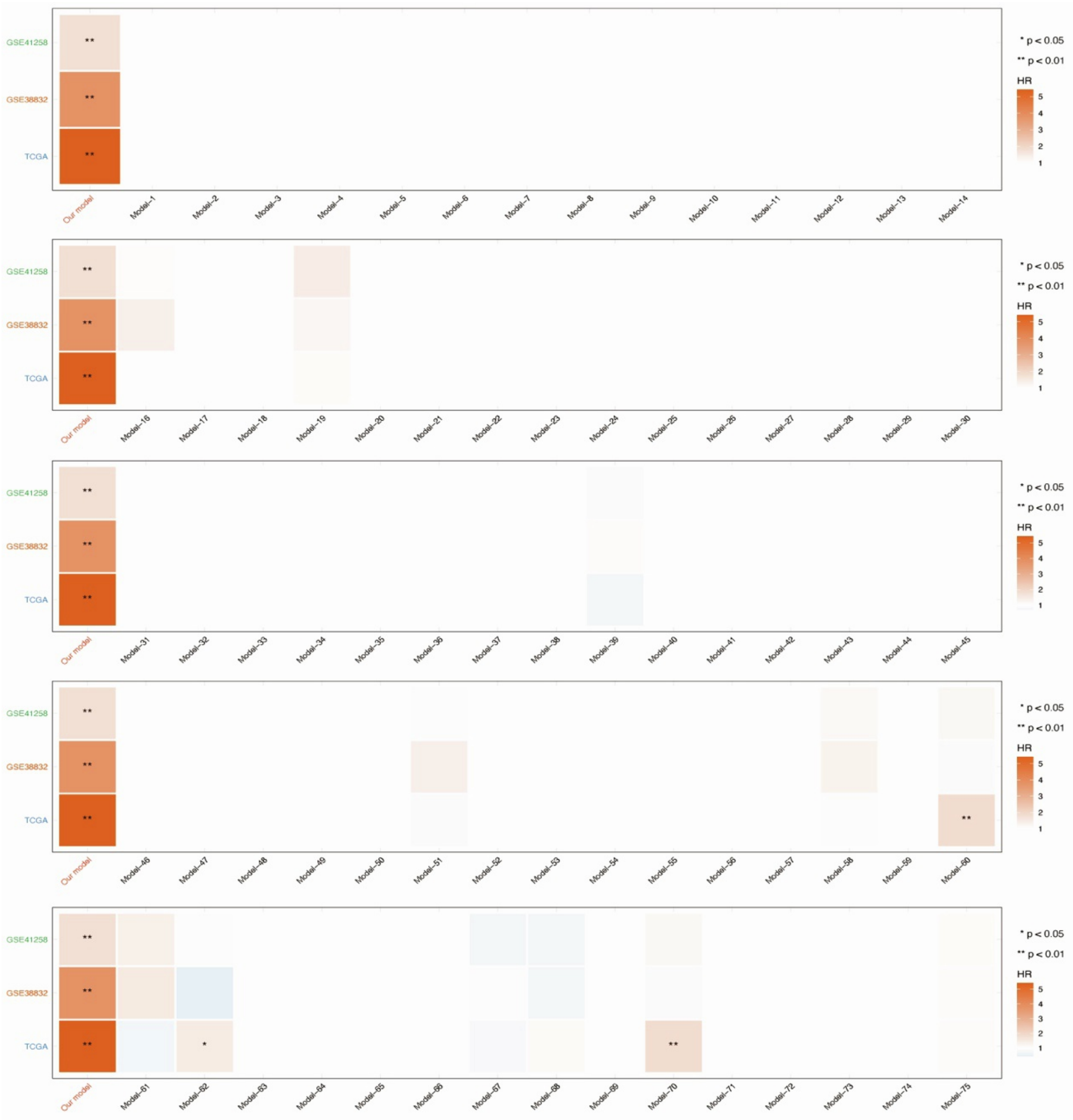

Figure S2. Comparison of hazard ratios between the StepCox[both]+GBM model and 74 other prognostic models, related to Figure 3.

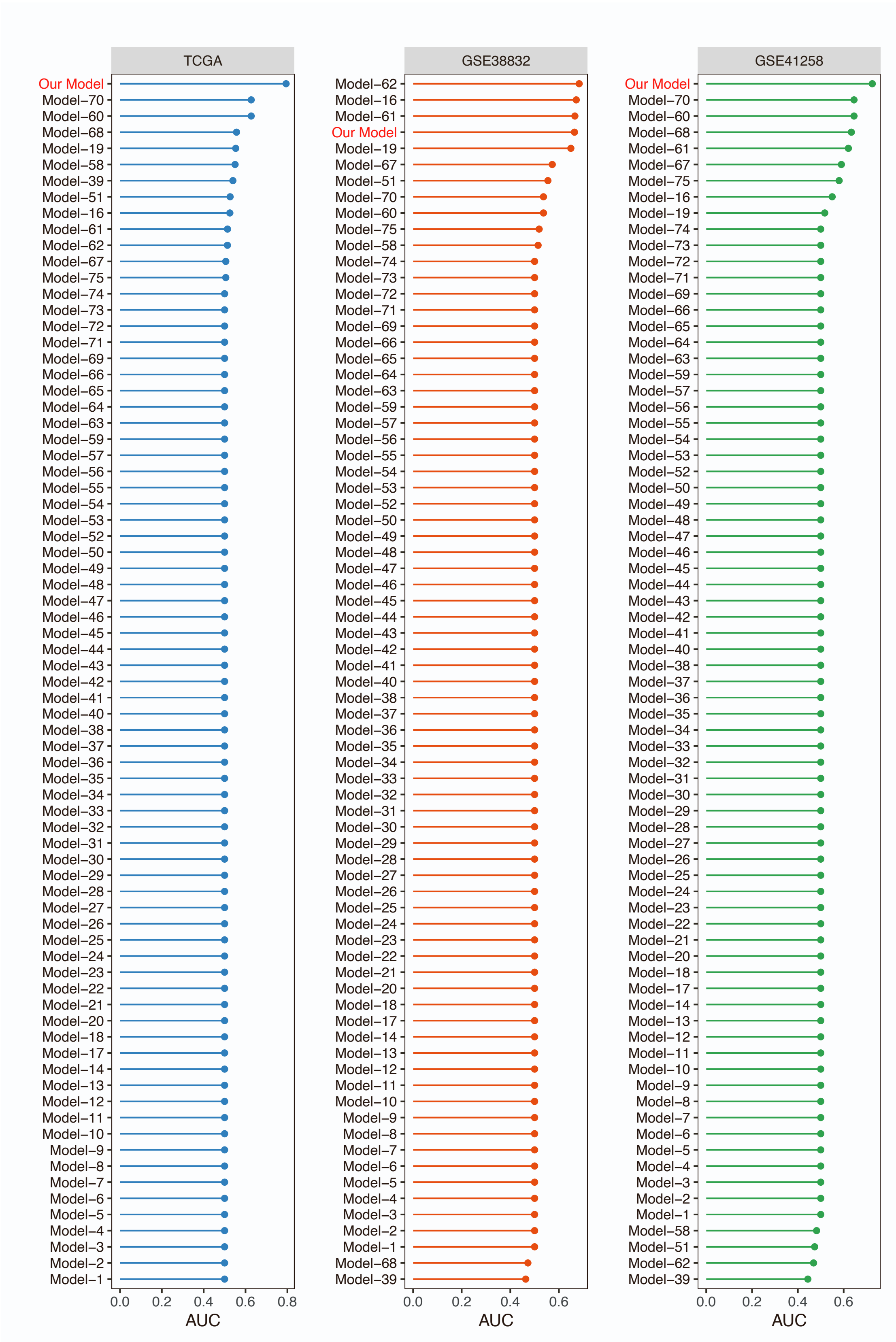

Figure S3. Specificity and sensitivity in predicting 1-year overall survival for the StepCox[both]+GBM model versus 74 existing models, related to Figure 3.

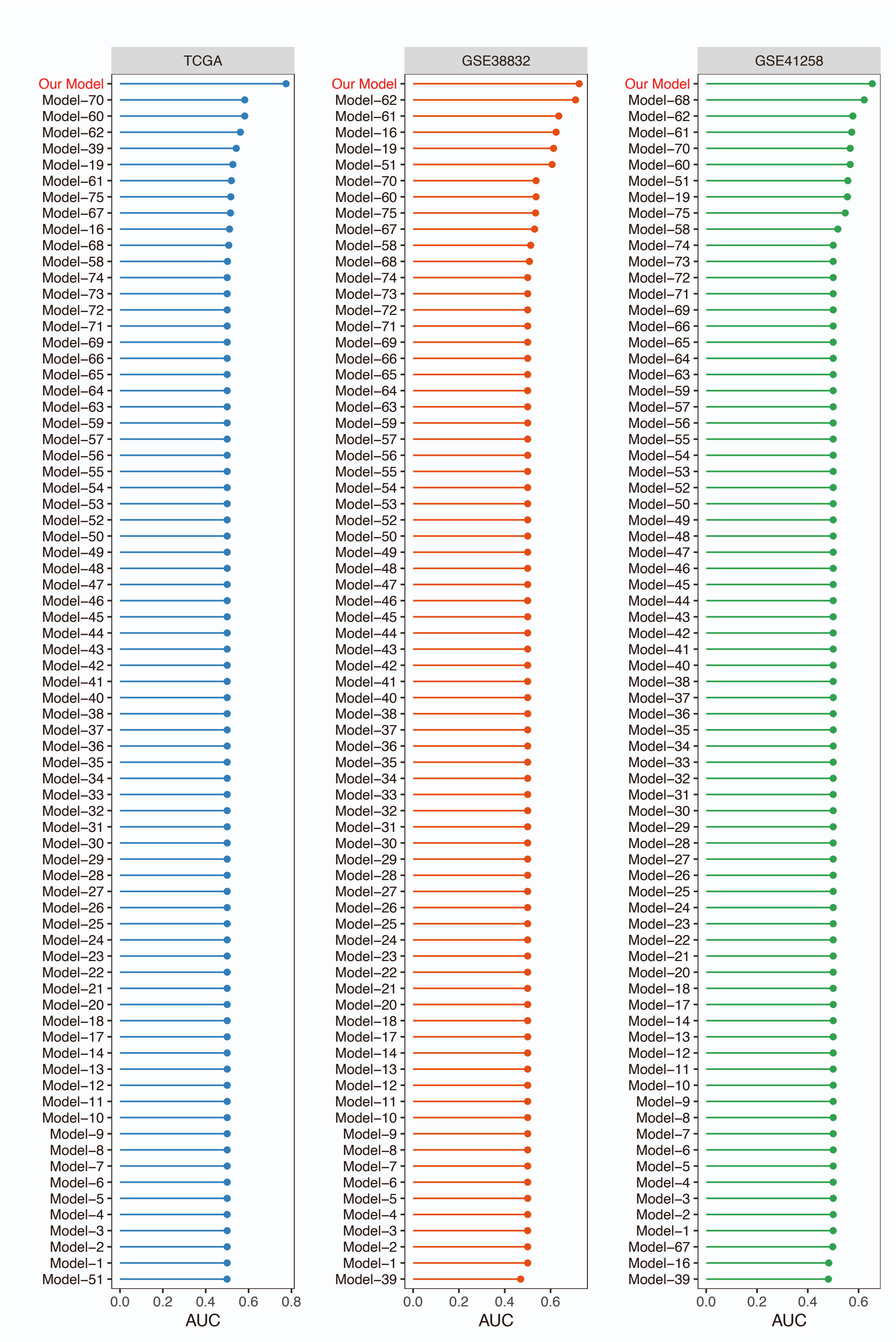

Figure S4. Specificity and sensitivity in predicting 3-year overall survival for the StepCox[both]+GBM model versus 74 existing models, related to Figure 3.

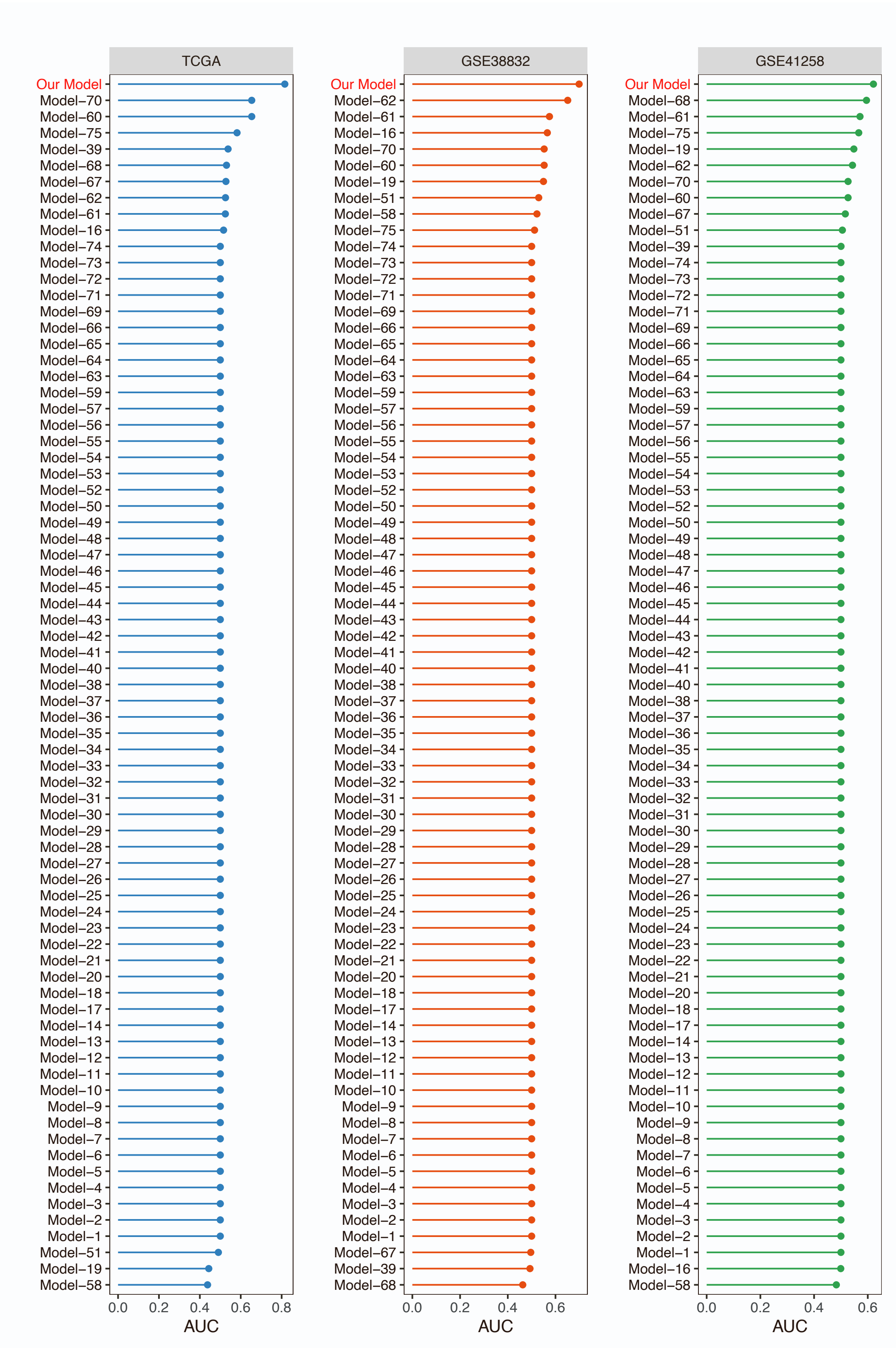

Figure S5. Specificity and sensitivity in predicting 5-year overall survival for the StepCox[both]+GBM model versus 74 existing models, related to Figure 3.

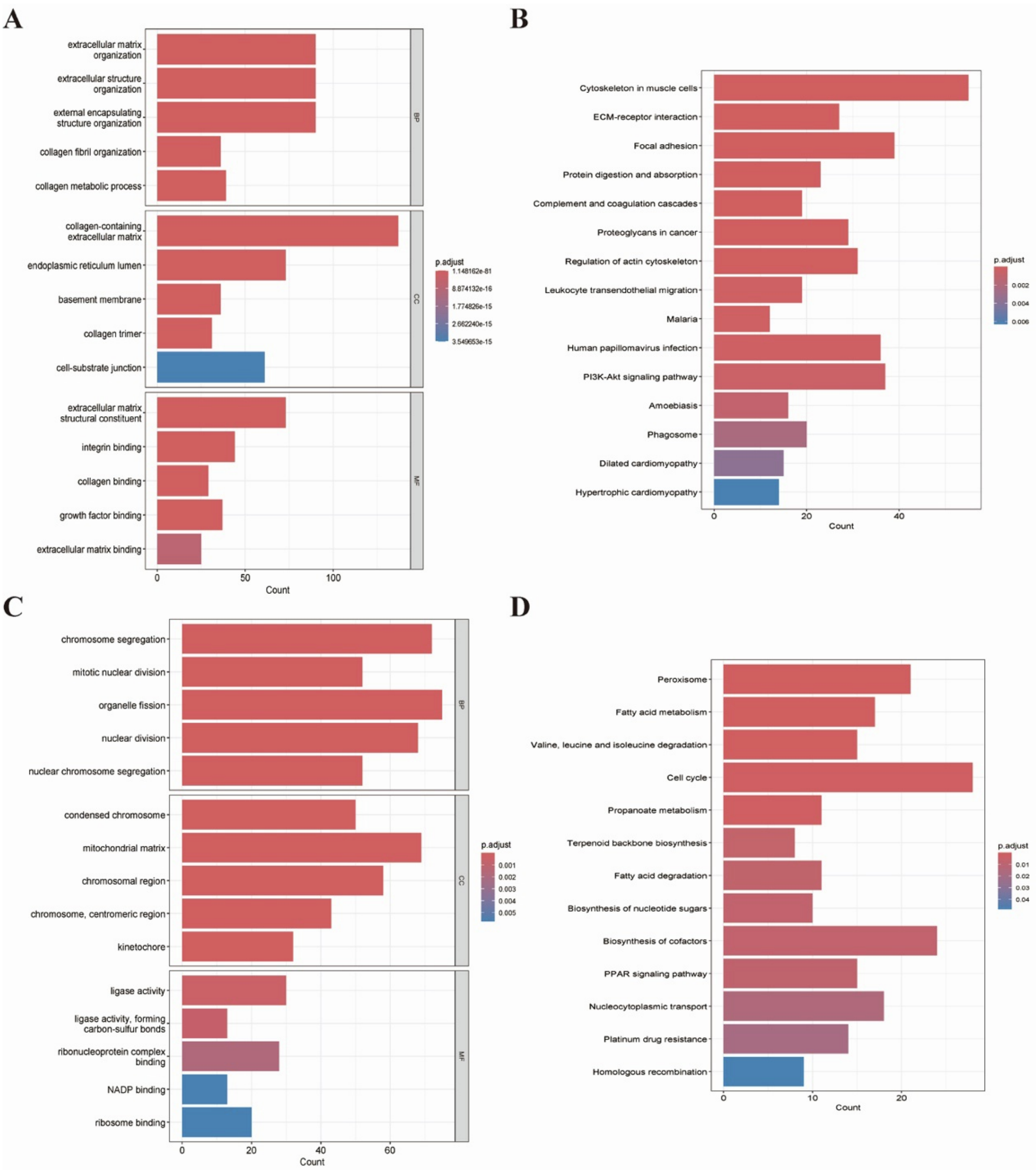

Figure S6. Transcriptomic characteristics of the high- and low-risk groups in the TCGA-CRC cohort, related to Figure 3.

(A) Bar plot showing significantly enriched GO terms in the high-risk group. (B) Bar plot of KEGG pathway enrichment analysis in the high-risk group. (C) Bar plot showing significantly enriched GO terms in the low-risk group. (D) Bar plot of KEGG pathway enrichment analysis in the low-risk group.

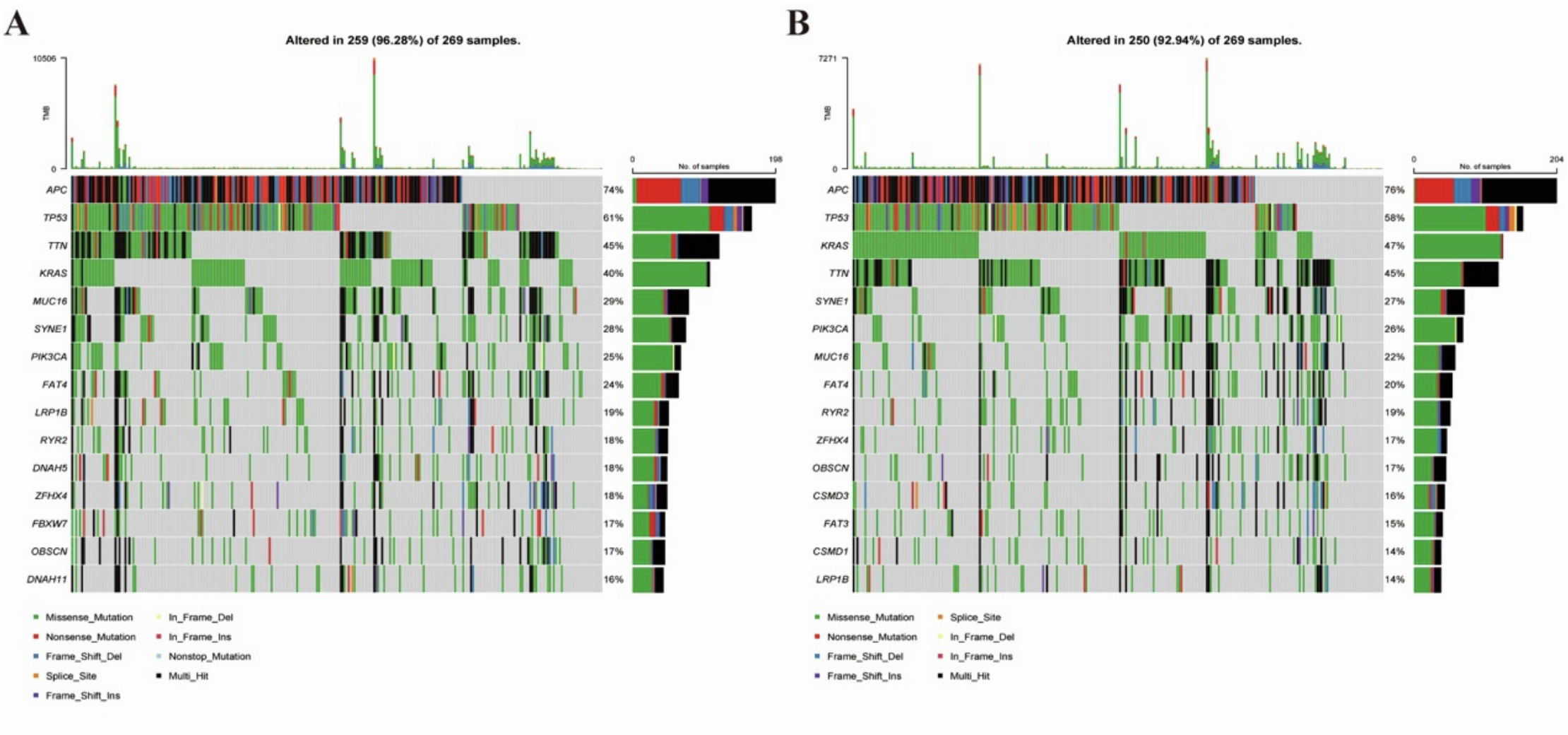

Figure S7. Mutational landscape of the high- and low-risk groups in the TCGA-CRC cohort, related to Figure 3.

(A) Waterfall plot depicting the gene mutation profile in the high-risk group.

(B) Waterfall plot illustrating the gene mutation profile in the low-risk group.

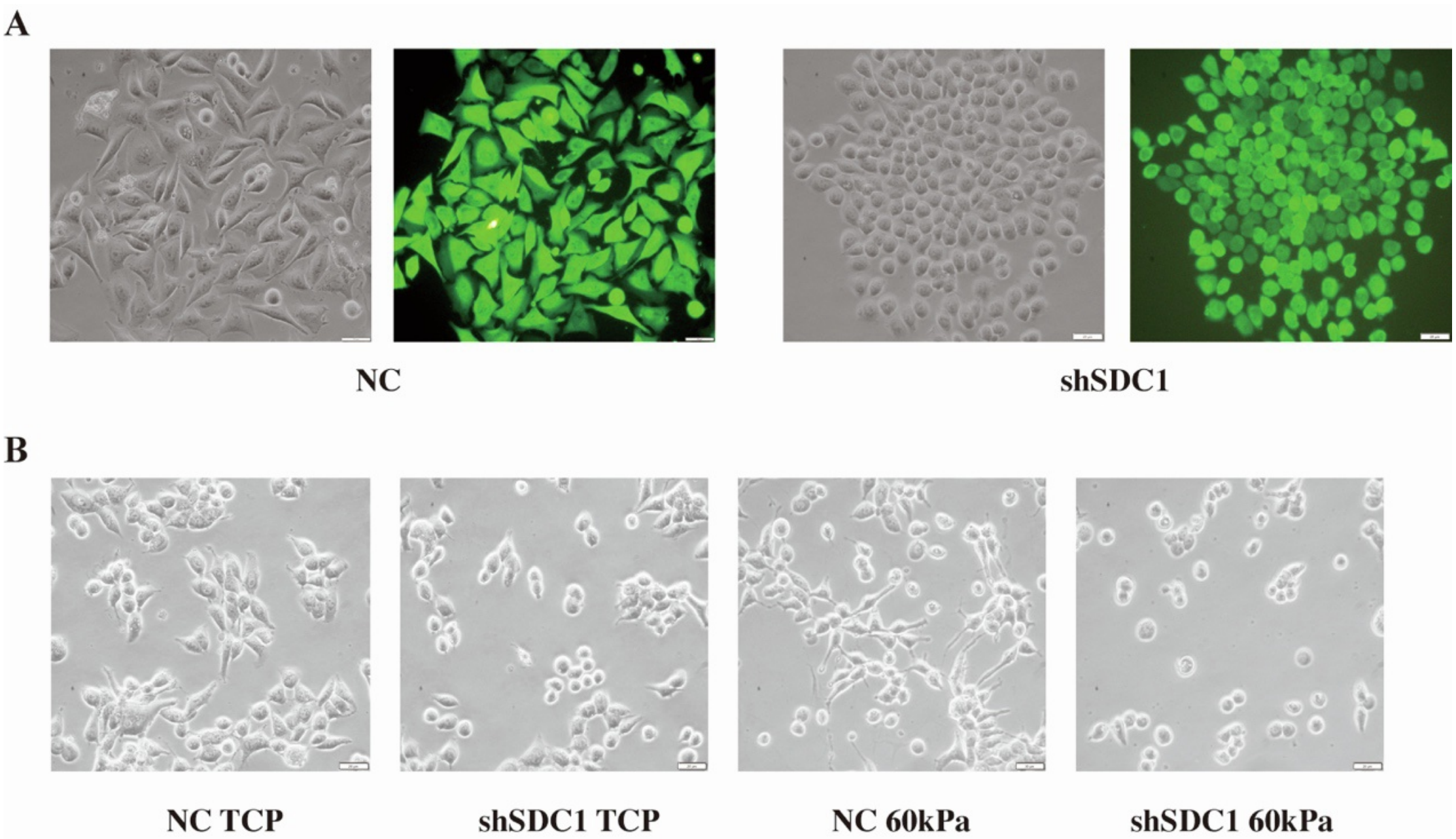

Figure S8. Fluorescence and bright-field images of DLD-1 cells after SDC1 knockdown and under different stiffness conditions, related to Figure 9.

(A) Representative bright-field and GFP fluorescence images of NC and shSDC1 DLD-1 cells. GFP fluorescence confirmed efficient transduction. Compared with NC cells, shSDC1 cells showed evident morphological changes, including a more rounded and clustered appearance.

(B) Representative bright-field images of DLD-1 cells under NC TCP, shSDC1 TCP, NC 60-kPa, and shSDC1 60-kPa conditions. The images show morphology differences associated with SDC1 knockdown and matrix stiffness. Scale bars, 20 μm.

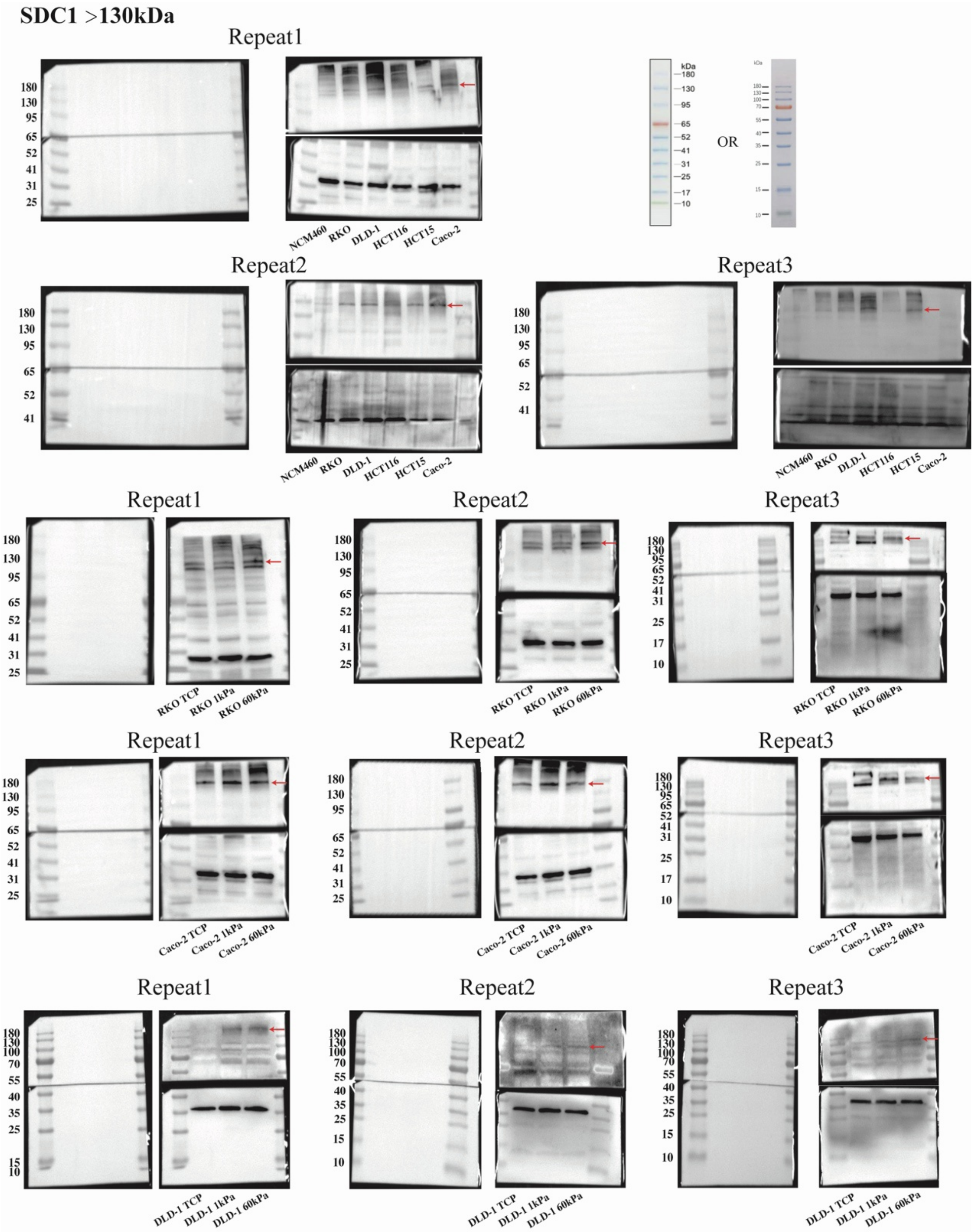

Figure S9. Full, uncropped western blot membranes related to Figure 8A and B, with three independent experimental replicates shown. Lane labels indicate the corresponding cell lines or stiffness conditions. Red arrows indicate the target bands used for quantification.

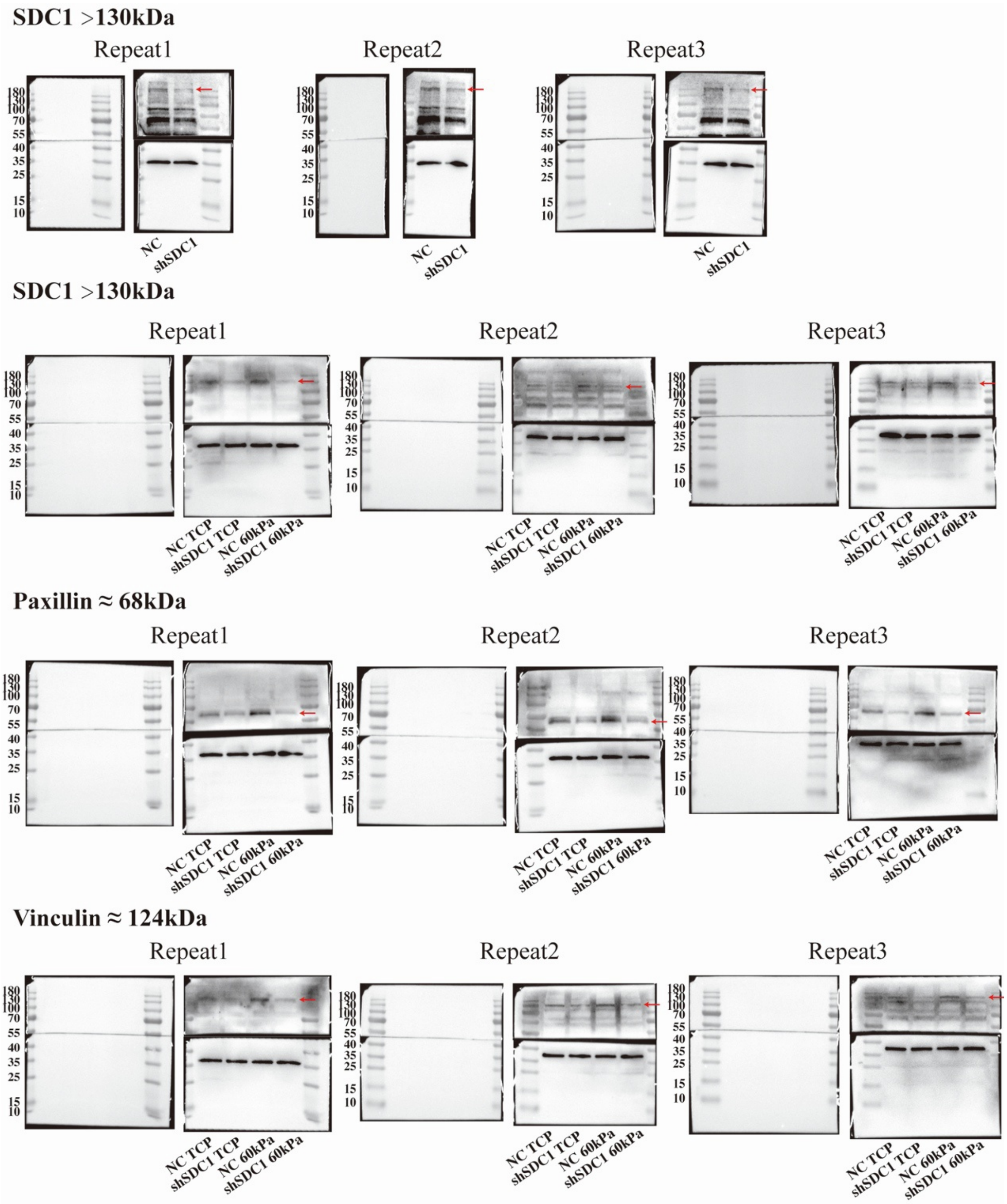

Figure S10. Full, uncropped western blot membranes related to Figure 9B and G, with three independent experimental replicates shown. Lane labels indicate the corresponding experimental groups. Red arrows indicate the target bands used for quantification.

Table S1. Primer sequences used for qRT-PCR, related to Figures 7 and 9 and STAR Methods.

Genes	Forward primer （5’-3’）	Reverse primer （5’-3’）
SNX7	ACTAAGACATCTCGTGGGGAA	CAGAGTGGGGTGTGCTTCTTC
SDC1	ACGGCTATTCCCACGTCTC	TCTGGCAGGACTACAGCCTC
CDCP1	GGCCCATGTCCTTTTGGGG	GCAGCTCTAAACCGATGCTCT
PTGES	TCCTAACCCTTTTGTCGCCTG	CGCTTCCCAGAGGATCTGC
EMP2	GTGCTTCTTGCTTTCATCATCG	TGCAATTCGTGTTGTTGGTACA
CALB2	ACTTTGACGCAGACGGAAATG	GAAGTTCTCTTCGGTTGGCAG
GAPDH	ACAACTTTGGTATCGTGGAAGG	GCCATCACGCCACAGTTTC

Table S2. shRNA sequences used for SDC1 knockdown, related to Figure 9 and STAR Methods.

shRNA	Target sequence (5′–3′)
Negative control	GTTCTCCGAACGTGTCACGTT
shRNA-1	GACTGCTTTGGACCTAAAT
shRNA-2	GAGCAGGACTTCACCTTTGAA
shRNA-3	CCGCAAATTGTGGCTACTAAT

Table S3. List of 323 differentially expressed genes common to both 2 kPa and 55 kPa substrate conditions, related to Figure 1 and STAR Methods.

Gene Name									
CCBL2	DHDDS	SFSWAP	TAC4	C2orf88	LINC00616	MCM4	CSDE1	DLST	BID
DESI2	OMA1	NEDD1	TRIM65	TMEM214	FAM200B	HGSNAT	EDEM3	DCAF4	ECT2
EIF4G3	SMIM12	LETMD1	SYNRG	C2orf54	NDUFC1	SLC39A14	RALGPS2	AAGAB	NGLY1
GNPAT	PANK4	NOP2	ALG13	PPIL3	WFS1	GSR	TRAF5	ATXN2L	ZMAT3
MIER1	CREM	UBE3B	ARRB2	CHN1	FCHO2	GRINA	CTNNBIP1	UBE2I	MLF 1.00
TMEM183A	OPTN	LRRC23	THRA	SPATS2L	PDLIM7	RALGDS	NFYC	ABR	MTMR14
TP53BP2	PITRM1	CENPJ	LIG3	TANC1	POC5	TUBB4B	OSBPL9	COPS3	MLH1
TRNT1	ABLIM1	DLEU2	KANSL1	RRBP1	ANKHD1	RMI1	SETDB1	PEMT	RPL22L1
RWDD3	BMI1	LAMP1	NEDD4L	RTEL1	HIST1H2AJ	SPTAN1	LBR	PRPSAP2	OCIAD1
DIO1	ZNF33A	KATNAL1	ZBTB14	EDEM2	MTHFD1L	RABEPK	USP24	TOB1	HSD17B4
EPS8L3	CASP10	NAXD	ATP5SL	AAR2	HIST1H3A	ODF2	ARHGEF2	MAP2K3	NSD1
SNX7	SPPL2A	DGKH	DEDD2	TSPO	HIST1H4A	AIF1L	CAPZB	EPN3	FOXP4
PLEKHA5	TCF7L2	CHD8	LDLR	PRR34	EXOC2	CCDC183	LGALS8	KCTD15	CYB5R4

C1orf43	DCLRE1C	RPPH1	AKAP8	PEX26	TJAP1	DIAPH2	NBL1	WDR62	CITED2
ADAR	ANK3	DCAF5	UBXN6	DGCR8	SCAF8	MORF4L2	RNF115	TJP3	PEX7
ZNF695	ANKRD26	TTLL5	PLEKHA4	EWSR1	TTK	SMS	MCMBP	ZNF181	GMNN
MTOR	TSPAN4	L2HGDH	SMARCA4	GGT1	TBC1D7	AKAP17A	ASB13	ZNF414	FIG4
SLC50A1	KDM2A	KLC1	CSNK1G2	TSEN2	SUMF2	MID1	USP6NL	PLEKHB2	ZNF655
RGS5	CEP164	TRAF3	BCKDHA	MYD88	DTX2	TBC1D8B	FAM213A	STEAP3	PMPCB
ARNT	FAM76B	POLE2	ZNF433	NISCH	CCDC136	ARMCX5	TBRG1	LTBP1	PTPN12
EFCAB2	ZNF215	MORF4L1	SAFB	VIPR1	IMPDH1	TIMM17B	RASSF7	CCT7	TNPO3
TARBP1	C11orf80	CSK	PLPPR2	EIF4A2	CLDN12	ERMAP	SLC3A2	TMEM177	MAFK
AP3B2	C11orf68	DMXL2	ZNF700	MITF	LOC441242	PIAS3	NFYB	SERPINE2	STEAP2
RAB3B	APLP2	AP4E1	GATAD2A	PIGX	VAMP7	AGL	LIMA1	MAP2	ASNS
MOSPD2	TBCEL	RRN3P1	LONP1	PDHB	MEST	CSRP1	TXNRD1	ASXL1	PUF60
PUM1	KLC2	NDE1	GPR108	ZNF589	DMTF1	EPB41	PPHLN1	MCM8	LETM2
HIPK1	CAPN1	NOL3	CHAF1A	MFSD1	SUN1	EFNA1	ZNF664	NCOA3	CHRAC1
ORC1	NADSYN1	PIGQ	ANKRD36	BBX	RNF216P1	CCDC18	ZNF26	PHACTR3	PHF2
LRRC39	DGKZ	PHKB	GALM	RPP14	LMTK2	ARID4B	CDK2AP1	SCAF4	TNFSF15
ADAM15	UBE4A	SDF2	LIMS1	B3GALNT1	PTK2	RERE	ANAPC7	PSMG1	HCCS
PHACTR4	GANAB	KAT2A	ASAP2	GXYLT2	TONSL	STMN1	TRMT5	PDE9A	SLC9A6
YY1AP1	EP400NL	LLGL2	USP39	CNOT10	ZNF251	APH1A	PPM1A	BCL2L13	STK26
MAPKAPK2	LRRC75A-AS1	LOC100630923							

Table S4. Co-expressed genes associated with liver metastasis, related to Figure 2 and STAR Methods.

Gene Name									
AZGP1	DDR1	GPRC5A	CALB2	DSC2	LIPH	TNFRSF21	SCNN1A	EPS8L1	GNG12
NBL1	SNX7	S100A16	BMP4	BCAR1	ITGB8	PTPRF	YAP1	USH1C	TNNC2
FKBP10	KRT7	PRKCDBP	ELF3	SPINT1	NT5E	TINAGL1	DSG2	C2orf82	TSPAN15
HOXA10	DST	LAMB3	EFNB2	ONECUT3	SOCS6	GJB3	KCNK1	CBLC	PTK6
TSPAN1	CAV1	KLK6	MALL	F2RL1	RIPK4	CDCP1	FARP1	NCKAP1	IRF6
NR2F2	RRM2	RHOD	KLK10	ITPKC	LAD1	PTGES	SEMA3C	CYP2W1	NPSR1
EPB41L1	F3	COL17A1	HMGA2	C6orf132	PITX1	EHF	SERINC2	LURAP1L	AGRN
STEAP1	CALB1	CDH1	NET1	TJP1	SDC1	SOWAHC	LMO7	UACA	HOXA9
SOX9	TM4SF1	LAMA3	CYR61	CDKN1C	STEAP2	IL20RA	PLEK2	AP1M2	PTK2
EFNA1	KRT8	SFN	KRT17	MYEOV	BCAM	ESRP1	OVOL1	MISP	MACC1
H1F0	KRT18	CCND1	ITGB4	RHOV	SERPINE2	MET	LETM2	RTKN	PPP1R14D
SUN1	KRT19	SLPI	PLPP2	EPS8	RAB25	EDN1	EPHA2	B3GNT3	SLC9A3R2
IGF2BP2	RND3	FOSL1	FHL2	EMP2	PPP1R13L	SULT2B1	CLDN1	CHMP4C	PLS3
ANXA3	CAV2	DIO3	PKP3	HYAL2	FAM213A	ARHGAP32	FAM83H	VWA1	MEST
ITGA3	PLAU	JUP	PPIC	LAMC2	SERPINB5	LMTK3	FAM129B	HOXB9	MYO10
LDLR	DCBLD2	AKAP12	CTTN	GCNT3	TPD52L1	TNKS1BP1	MLPH	S100A14	PCDH1
MPZL2	CLDN4	AHNAK2	PRSS22	CAMK2N1	SEMA3B	PLCD3	CDC42BPB	UBE2C	SOD3
PLEKHA5	TACSTD2	KRT6B	DSP	BAMBI	CRB3	ITGB5	RP11-54H7.4		
